# Theta Power at 10 Months of Age Predicts Developmental Change in Language in Infants With and Without an Elevated Likelihood for Autism

**DOI:** 10.1111/desc.70194

**Published:** 2026-04-13

**Authors:** Eirini Papageorgopoulou, Jannath Begum Ali, Greg Pasco, Amy Goodwin, Luke Mason, Mark H. Johnson, Tony Charman, Kalinka Timmer, Emily J. H. Jones, STAARS team

**Affiliations:** ^1^ Centre for Brain and Cognitive Development, School of Psychological Sciences Birkbeck, University of London London UK; ^2^ Faculty of Psychology University of Warsaw Warsaw Poland; ^3^ Institute of Psychiatry, Psychology & Neuroscience Department of Psychology King's College London London UK; ^4^ Department of Forensic and Neurodevelopmental Science King's College London London UK; ^5^ Department of Psychology University of Cambridge Cambridge UK; ^6^ Department of Child and Adolescent Psychiatry King's College London London UK; ^7^ MRC Centre for Neurodevelopmental Disorders King's College London London UK

**Keywords:** autism spectrum disorder, EEG power, language

## Abstract

Infants with an Elevated Likelihood (EL) for autism are more likely to experience language delays, but their language developmental trajectories are highly heterogeneous. The neurodevelopmental processes driving this variability remain unclear, yet understanding them is important for parsing the heterogeneity in language and informing earlier intervention. However, no studies have examined how developmental changes in brain function are related to changes in language ability across time in this population. In this prospective longitudinal study, we investigated the developmental associations between electroencephalography (EEG) and language measures in a sample of infants enriched for varied language outcomes: Infants with EL and Typical Likelihood (TL) for autism (EL = 99, TL = 60). Measures of EEG power in the relative frontal theta and alpha ranges during natural viewing of social and nonsocial naturalistic videos and observer assessment of language ability were collected at 10, 14, 24 and 36 months. As expected, the EL and TL groups differed in the slope of language ability, with the EL group showing less steep increases in language compared to the TL group. Further, 10‐month theta power (but not alpha) was a significant predictor of developmental change in language; autism likelihood group did not moderate this association and theta development was not related to language. Overall, findings suggest slowed language development as an autism trait symptom and that theta power is a predictor of general language ability, not specific to autism. Future research should consider incorporating additional measures to examine the potential environmental or genetic contributions to these associations.

**Summary:**

- Infants with Elevated Likelihood (EL) for autism vary in their language development, while the neurodevelopmental processes driving this variability are still unclear.
- The relation between developmental changes in relative electroencephalography (EEG) spectral power and language ability across 10–36 months in infants with and without an EL for autism was examined.
- The EL group showed less steep increases in language development compared to the TL group, but the groups did not differ in relative EEG power.
- Theta power was a significant predictor of developmental change in language; this association was not moderated by the autism likelihood group.

## 1 Introduction

Autism is a neurodevelopmental condition that can be diagnosed from around 2 to 3 years of age based on social communication difficulties, restrictive interests, repetitive behaviours and sensory anomalies (American Psychiatric Association 2013). Language delays are one of the most common co‐occurring patterns associated with early autism diagnosis (Tager‐Flusberg 2016). In fact, language and communication difficulties are among the most frequent concerns expressed by parents early in toddlerhood before a formal diagnosis of autism takes place (Chawarska et al. 2007; Herlihy et al. 2015). Given the high heritability of the condition (Hansen et al. 2019; Tick et al. 2016), infant siblings of an older autistic child have an increased likelihood of developing autism themselves (thereby infants with Elevated Likelihood, EL) and many may exhibit language‐related difficulties (Jones et al. 2014; Ozonoff et al. 2011). Indeed, several prospective longitudinal studies, where EL groups are assessed from early infancy to toddlerhood, have shown that infants with EL who have subsequently been diagnosed with autism (EL‐autism) exhibit delays in both language production and comprehension (Estes et al. 2015; Mitchell et al. 2006). Specifically, after the first year of life, delays in pre‐speech vocal behaviour and production or comprehension of fewer single words and phrases are observed in EL‐autism infants compared to infant siblings of an older neurotypically developing child (thereby infants with Typical Likelihood, TL) and to those with EL with no autism outcome (EL‐no autism) (Mitchell et al. 2006; Zwaigenbaum et al. 2005). However, the mechanisms underlying altered language developmental trajectories in this population are yet to be clarified.

Even though language delays are among the most common early markers of autism, how these delays unfold over time remains unclear, especially given the substantial heterogeneity in language development. Firstly, trajectories of language development vary among autistic individuals, encompassing language profiles of lower or faster improvement, regression, or a plateau (Anderson et al. 2007; Pickles et al. 2014; Tager‐Flusberg and Kasari 2013). For instance, Pickles et al. (2022) found that autistic preschoolers with language regression showed later delays in expressive communication, but regardless of regression status, some developed age‐appropriate or advanced expressive skills by age 10. Smith et al. (2007) revealed four different profiles of expressive language development in autistic children across 2 years. Latreche et al. (2024) identified three canonical language profiles in preschool children, each with distinct developmental patterns: children without language impairments showed a shifted trajectory compared to neurotypicals, while those with language impairments exhibited a slower rate of acquisition, reflecting both delay and slowdown. Secondly, early signs of language difficulties are not always observable in EL‐autism infants and can also be present in those with EL who may not receive a later diagnosis (Hudry et al. 2014; Marrus et al. 2018; Messinger et al. 2013; Szatmari et al. 2016). At the same time, higher language skills early on contribute to better developmental outcomes in autistic children (Mawhood et al. 2000; Pickles et al. 2022). Thus, examining language developmental trajectories at several time points in the first years of life in the context of autism is of great importance for understanding language variability, identifying any longer‐ and shorter‐term changes in skill and informing early targeted interventions to promote language development.

Understanding language development in autism may require examining functional brain development. Several factors (genetic and others) related to autism susceptibility and co‐occurring conditions, such as language delay, have been hypothesised to drive alterations in the early functional and structural organisation of brain networks. Altered development of neural systems may, in turn, alter infant opportunities for social learning from their environment, affecting infant social behaviour and contributing to an atypical developmental route (Elsabbagh 2020). Substantial evidence for altered trajectories of brain development in infants at EL is evident very early, before the emergence of overt behavioural symptoms, with brain function measures, such as those collected using electroencephalography (EEG) distinguishing TL and EL infants earlier than behavioural measures, within the first year of life (Elsabbagh et al. 2012; Keehn et al. 2015; Key and Stone 2012; Levin et al. 2017; Peck et al. 2021; Tierney et al. 2012; Tye et al. 2020). In particular, early brain differences have been documented in studies using measures such as EEG spectral power. EEG spectral power reflects the summation of both broad band firing and synchronised neuronal firing, vital for self‐organisation of neural systems and cortical maturation (Uhlhaas et al. 2010). Differences in frontal EEG spectral power across different frequency bands have been observed from as early as three months between EL and TL infants (Gabard‐Durnam et al. 2019; Levin et al. 2017; Tierney et al. 2012), suggesting that frontal EEG power may be sensitive in identifying early brain alterations related to subclinical traits on top of autism diagnosis.

Several studies suggest associations between EEG spectral power and language skills in neurotypical and atypical development (Levin et al. 2017; Lum et al. 2022; Pierce et al. 2021; Wilkinson et al. 2020). In general, alpha and theta power might be related to a broader proficiency in language. For instance, alpha oscillatory power has been linked to speech processing guided by attentional control (Wöstmann et al. 2017), and theta power has been shown to be modulated by infant‐directed speech, reflecting social attention engagement of the child to the mother (Orekhova et al. 2006). In turn, both alpha and theta oscillations have been linked to verbal fluency (Wojtecki et al. 2017). Therefore, both may be informative in delineating the variability in language development in autism, but, do date, the findings have been inconsistent. A study by Levin et al. (2017) demonstrated that reduced frontal alpha power, but not theta, at 3 months was linked to lower expressive language scores measured through the Mullen Scales of Early Learning (MSEL) at 12 months in infants at EL for autism. However, associations did not remain between 3‐month alpha power and expressive language at 2 and 3 years of age. Similarly, in a more recent study comparing EL and TL infant siblings, EEG power across a range of frequency bands at 6 months was investigated in relation to concurrent (6 months) and developmental change (up to 36 months) in language ability through the MSEL (Huberty et al. 2023). Although no associations were observed between theta power and language, the results revealed positive links between the alpha frequency band and concurrent expressive language skills. However, this association was not moderated by sibling group regardless of the EL group having generally lower language scores. Other research that focused on the trajectories of EEG power in several frequency bands across the first 2 years in relation to a specified language outcome at 24 months showed a relationship between theta and gamma power and language scores (Wilkinson et al. 2020). The results also showed increased 6‐month theta power consistently related to lower language skills, specifically in the EL group, with and without controlling for sex and parental education. In addition, Jones et al. (2020) found a positive relationship between 12‐month changes in frontal theta power when viewing dynamic semi‐naturalistic videos and verbal/non‐verbal ability at 24 months in infants at EL. A marginally significant positive association was also found with 36‐month verbal skills, which varied by outcome group (EL‐autism, EL‐no autism and TL) but was significant for the group who later developed autism. Overall, these studies are particularly relevant to the current work as they provide an indication that early EEG patterns, specifically theta and alpha power, are associated with language outcomes, especially in infants at EL for autism. However, the results above show some inconsistencies between theta and alpha power and language which could be due to the different time points or methodology used in each study. Nevertheless, they suggest that EEG spectral power in these frequency bands warrants further investigation as a candidate endophenotype that could help understand the mechanisms that underlie individual variation in language skills in infants at EL for autism.

Although prior studies offer essential insights into the neural processes underlying language skills, more research is needed to consolidate these findings. Most of the research has been cross‐sectional or has examined one domain across time and how its trajectory links to a domain measured at a single time point, which might preclude our understanding of the developmental course of both brain function and language skills in infants at EL. As previously mentioned, language developmental trajectories in EL siblings have considerable variability. In addition, EEG power in this population appears to change dynamically with age (Gabard‐Durnam et al. 2019; Tierney et al. 2012), which may explain the inconsistencies in the literature between alpha and theta power in relation to language ability. Examining the trajectories of EEG power changes may be more robust as a candidate endophenotype of risk for language impairment than a single time point. In this study, we aimed to investigate the developmental changes in language skills and their possible neural underpinnings by examining how language relates to brain function changes in a sample of infants at EL and TL for autism. To our knowledge, this is the first study to investigate the relationship between infant neural activity and language ability over developmental time in a period of dramatic brain and language growth, which may offer a more complete picture and understanding of the variability in brain and language development as well as illuminate the possible developmental processes linked to later language outcomes in populations with an elevated familial likelihood of autism.

Sex and maternal education were also considered in this study due to evidence linking both factors to early brain development and language outcomes. For instance, males are at higher likelihood for language delays and developmental language disorder, and show distinct early neural patterns, including greater theta power in infancy (Chilosi et al. 2023; Jones et al. 2015). Maternal education has also been associated with variability in infant EEG power, including the alpha band (Pierce et al. 2019) and with early language development, with higher education levels generally linked to stronger language comprehension skills in children (Zambrana et al. 2012). Therefore, controlling for sex and maternal education helps account for meaningful sources of variance in both neural and language development trajectories.

### 1.1 Aims

The current study aimed to examine EEG spectral power responses to ecologically valid, dynamic social and non‐social stimuli, and their links to language ability across 10 and 36 months in a sample of infants at EL and TL for autism. Relative EEG power in the theta (3–6 Hz) and alpha (6–9 Hz) bands was used as these bands have previously been associated with measures of language ability in infant siblings. We chose to examine EEG power to both social and non‐social videos (representing brain responses to averaged social and non‐social stimuli) as they matched more closely to a child's normative environment. We particularly investigated (1) associations between EEG frontal power and language ability at 10 months, (2) the links between developmental change in both EEG power and language ability over time, (3) and whether EEG power/language at 10 months was related to developmental change in language/EEG power, respectively. We hypothesised that we would see a relationship between frontal alpha power at 10 months and concurrent, but not developmental change in language ability, consistent with prior evidence. Given the inextricable link between brain development and behaviour, we hypothesised that developmental change in EEG power would be related to developmental change in language skills. Crucially, we also expected that autism likelihood groups would be significant predictors of EEG power and language development. We ask whether these groups would differ in the relationship between EEG power and language. We hypothesised that the autism likelihood group would significantly predict the trajectories of EEG power and language ability but we did not expect associations between EEG power and language to differ by autism likelihood group, given prior evidence of nonsignificant differences by EL and TL group. We controlled for infant biological sex and maternal education level as they could potentially confound the relationship between brain function and language (Chilosi et al. 2023; Jones et al. 2015; Pierce et al. 2019; Zambrana et al. 2012).

## 2 Methods

### 2.1 Participants

A total of 161 infants were recruited in a longitudinal study from 2013 to 2019 [Study name and location concealed]. Infants had either a first‐degree relative with a community clinical diagnosis of autism (infants at EL) or a first‐degree relative with neurotypical development (infants at TL). The parents gave informed written consent before their children participated in the study. [Research ethics and name of university concealed]. Reimbursement of travel expenses or subsistence and overnight stay was provided to the families who participated, if necessary. After each visit, the infants were given a t‐shirt and a certificate.

The infants were followed during four visits: at 10, 14, 24 and 36 months. For the assessment of language ability, 152 (91.6%) infants participated at 10, 142 (85.5%) at 14, 129 (77.7%) at 24 and 120 (72.3%) at 36 months. These time points were selected as the period when differences in language skills begin to become apparent in infant siblings, while a diagnosis can be made around 24 and 36 months. This period is also characterised by increasing brain and language growth, which is sensitive to language learning and acquisition. In addition, group differences have been shown in EEG power before the first year, though no study has investigated these and their links to language, including the 10‐ and 14‐month time points. For the electrophysiological data collection, 139 (83.7%) infants participated at 10, 110 (66.3%) at 14, 105 (63.3%) at 24 and 107 (64.5%) at 36 months. Participants who provided data for at least one time point for language and EEG spectral power were included in the analysis, as missing data were accommodated within the chosen analytical method (see ‘analytical approach’ section below). Of the 161 infants participated, six had missing EEG data due to technical issues (*n* = 4) or due to absence from the lab visit (*n* = 2) (155 in total). In language, two infants had missing MSEL data due to absence from the lab visit (159 in total). Our final sample included 159 participants as there were enough data available for them according to the analytical method accounting for missing data. In the models examining the effect of autism likelihood group as a covariate, three infants were half‐siblings and excluded from the analyses.

We also examined data availability across the four time points separately for language and EEG (alpha and theta power) measures. For the language data, 103 participants (64.8%) had data available at all four time points, 30 participants (18.9%) had data at three time points, 15 participants (9.4%) had data at two time points and 11 participants (6.9%) had data at only one time point. For the EEG data, 68 participants (42.8%) had complete data across all four time points, 38 participants (23.9%) had data at three time points, 26 participants (16.4%) at two time points and 23 participants (14.5%) at only one time point.

Table 1 provides the participant demographic characteristics by autism likelihood group. Maternal education was categorised into four levels (primary, secondary, tertiary undergraduate, tertiary postgraduate) and treated as ordinal for the purpose of descriptive group comparisons. The two groups did not differ in ethnicity, biological sex and age at any visit. However, the groups significantly differed in maternal education (*p* = 0.012). The maternal education level in infants at TL was higher than that of infants at EL. The mean values of language development, theta power and alpha power are provided in Table 2 (refer to Figure S1 in the appendix for a depiction of the average trajectories of language, theta and alpha power).

**TABLE 1:** Demographic characteristics by autism likelihood group.

Characteristic	*n*	Total	*n*	TL	*n*	EL	*χ* ^2^/*t* (df)	*p*
Biological sex (Male/Female)^a^	156	88/68	57	35/22	99	53/46	0.91	0.216^a^
Maternal education (m, SD)	150	2.99 (0.77)	55	3.20 (0.78)	95	2.87 (0.75)	2.54 (148)	0.012
Age in days (m, SD)								
10 months	149	320.90 (17.79)	53	323.00 (22.60)	96	319.33 (14.54)	1.20 (147)	0.231
14 months	139	449.61 (19.33)	47	447.62 (20.96)	92	450.32 (18.73)	−0.77 (137)	0.442
24 months	128	767.93 (40.90)	46	763.91 (36.10)	82	770.13 (43.70)	−0.82 (126)	0.413
36 months	119	1144.7 (54.23)	42	1144.19 (69.51)	77	1144.88 (44.38)	−0.07 (117)	0.947
Ethnicity^a^ (*n* %)		154 (92.8)		59 (98.3)		95 (96.0)	3.47	0.516^a^
White		125 (75.3)		52 (86.7)		73 (73.7)		
Asian		9 (5.4)		3 (5.0)		6 (6.1)		
Black		2 (1.2)		—		2 (2.0)		
Mixed race		16 (9.6)		4 (6.7)		12 (12.1)		
Other		2 (1.2)		—		2 (2.1)		

Abbreviations: *t*, independent samples t‐test; *χ* ^2^, Chi‐squared test.

^a^Fisher's Exact Test was used due to low expected cell count.

**TABLE 2:** Mean scores of the language, theta power and alpha power variables at 10, 14, 24 and 36 months across the whole sample and by EL and TL group.

Variables	Total	TL group	EL group
	Mean (SD)		
Language 10	36.71 (9.25)	36.42 (8.38)	36.88 (9.74)
Language 14	34.18 (8.09)	35.57 (6.82)	33.47 (8.62)
Language 24	51.43 (12.09)	54.51 (10.93)	49.68 (12.43)
Language 36	54.42 (10.83)	58.90 (10.64)	51.91 (10.17)
Theta power 10	0.72 (0.06)	0.73 (0.06)	0.71 (0.06)
Theta power 14	0.68 (0.06)	0.70 (0.07)	0.68 (0.06)
Theta power 24	0.64 (0.06)	0.66 (0.06)	0.64 (0.06)
Theta power 36	0.63 (0.06)	0.63 (0.07)	0.63 (0.06)
Alpha power 10	0.16 (0.05)	0.15 (0.05)	0.16 (0.05)
Alpha power 14	0.18 (0.06)	0.17 (0.06)	0.19 (0.05)
Alpha power 24	0.19 (0.04)	0.19 (0.06)	0.19 (0.04)
Alpha power 36	0.19 (0.05)	0.19 (0.05)	0.19 (0.05)

*Note*: Language *T*‐scores were used to calculate mean language scores at each time point.

### 2.2 Measures

#### 2.2.1 EEG Collection and Processing

Electroencephalography was collected using the EGI NetAmps 400 amplifier and EGI (Philips Neuro, Oregon, USA) 128‐electrode Hydrocel Sensor Net, online referenced to Cz at 500 Hz at 10 months and 1000 Hz at 14–36 months. A bandpass filter (0.1–100 Hz) was applied, and EEG was 1‐s segmented. Data artefact rejection was done manually in NetStation 4.5. Segments with excessive artefacts, the infant paid no attention to the video, eye blinks, motor movement or segments with >25 noisy channels were excluded manually. Infants who had fewer than 10 artefact‐free trials in either condition were excluded (refer to Supporting Information 1, Table S1, for the mean number of valid trials by autism likelihood and outcome group). Noisy channels were interpolated from neighbouring channels using spline interpolation. 1‐s nonoverlapping segments were referenced to the average reference, imported into Matlab, detrended and subjected to a fast Fourier transform (FFT); with a 1 Hz resolution; values were extracted from 1 to 20 Hz. Power values were logged and averaged across artefact‐free segments and frequency ranges: theta (3–6 Hz) and alpha (6–9 Hz), within a priori topographical groups of electrodes (Figure S2). To confirm the appropriateness of the theta and alpha bands across the age range studied, we visually inspected average power spectra for each age group (10, 14, 24 and 36 months), scalp region (frontal, occipital) and condition (see below). Across all comparisons, the dominant peaks for theta and alpha activity consistently fell within the selected bands, supporting the validity of using fixed frequency ranges in this sample (Figure S3). Relative power was calculated as the sum (power in the relevant range)/sum (power across the full range specified), providing a proportion of absolute power within the theta and alpha frequency ranges. Relative power is thought to be more robust than absolute power in developmental populations and has shown higher test‐retest reliability, higher sensitivity to age‐related changes and less susceptibility to artefacts (Begum‐Ali et al. 2022; Benninger et al. 1984; Clarke et al. 2001; Govindan et al. 2017; John et al. 1980). Last, for the current study, frontal left, central and right frequencies were averaged for the theta and alpha bands at each time point.

##### 2.2.1.1 EEG Stimuli

During EEG recording, infants were seated on their parent's lap at 60 cm from a screen, presenting women singing nursery rhymes (dynamic social videos) or moving toys (dynamic non‐social videos). These stimuli were used for consistency with previous research (Haartsen et al. 2022; Jones et al. 2020) and because they represent ecologically valid stimuli that are closer to a child's natural environment than static stimuli. In addition, in previous studies using “baseline” EEG, recordings are often obtained while infants passively observe stimuli (i.e., an experimenter blowing bubbles), which is more naturalistic but much less controlled as it can vary significantly across participants and sessions. In contrast, our study employed a naturalistic video viewing task in which all infants viewed the exact same stimuli, providing a standardised and ecologically valid context. We consider this approach as an improvement of the other paradigms because it enhances experimental control while retaining naturalistic relevance and improves comparability across participants. The screen had a diagonal size of 23″ (58.42 cm × 28.6 cm, 52° × 26.8°, aspect ratio of 16:9). Social videos included two women showing their faces, torsos, and hands with corresponding gestures. The nursery rhymes were ‘Hi Baby, Where Are My Eyes?’, ‘Itsy Bitsy Spider’, ‘The Wheels on the Bus’, ‘Twinkle Twinkle Little Star’ and ‘Pat‐a‐cake’ (played in this fixed order). The non‐social videos showed spinning toys in motion, balls popping within a clear plastic toy and balls rolling down a chute, with complete absence of social content in these videos. Each video was 1 min long and presented up to three times, for 3 min. The order of the social/non‐social videos was counterbalanced for all the participants. Other visual tasks were presented between each block of videos but are not reported here. For the current study, frontal theta/alpha power to social videos was averaged with frontal theta/alpha power to non‐social videos at each time point, representing the frontal theta/alpha power to social and non‐social stimuli.

Further, to establish that the size of the stimuli was equal across participants in the event of technical problems with or changes in the monitor screen during the longitudinal study, stimuli presentation was undertaken within a ‘virtual window’ sized 17″ diagonally (34.5 cm × 25.9 cm, 32.1° × 24.4°, with a native resolution of 1280 × 1024 pixels and an aspect ratio of 5:4) with black borders around the screen. The display resolution of the stimuli was 37.1 pixels per cm. To maintain the source aspect ratio of 16:9 upon presentation within the ‘virtual window’, all the videos on the screen were scaled to 32.6 cm × 31 cm (30.4° × 29°, 1210 × 1150 pixels). Refer to Supporting Information 2 for further description of the experimental procedure.

#### 2.2.2 Behavioural Measure

The Mullen Scales of Early Learning (MSEL, Mullen 1995) is a standardised assessment of developmental ability. Trained experimenters administered it at 10, 14, 24 and 36 months. The MSEL contains scales focusing on expressive and receptive language, visual reception, gross and fine motor skills. The expressive and receptive language T‐scores were used for the current study. The expressive language scale contains 28 items assessing productive language ability, such as speaking, language formation and verbal conceptualisation. The receptive language scale contains 33 items and assesses how the child processes auditory linguistic input that measure comprehension, memory and sequencing. Refer to Supporting Information 3 for a description of the MSEL administration. Both scales were averaged for this study, providing an overall ‘language’ score at each time point. Since standardised language scores were used, an increase in language across time would represent an improvement in language skills relative to normative development. While T‐scores reflect age‐normed performance and are useful for comparing developmental trajectories across time, it is important to note that decreases in T‐scores do not necessarily indicate a loss of language skills. Rather, they may reflect slower than expected rates of development. Descriptive data on raw scores (Table S2) show that, overall, children's language abilities increased over time, indicating continued skill acquisition.

### 2.3 Analytic Approach

Latent growth curve modelling (LGCM) was used for data analyses of the current study (Bollen and Curran 2006). Latent growth curve modelling is a flexible approach for modelling linear or nonlinear change, considering individual differences in the trajectories of change. Before examining the links between language skills and relative frontal theta/alpha spectral power over developmental time, a series of LGCMs were run to test for the optimal functional form of growth separately for language, theta power and alpha power. The optimal functional form of growth was based on model fit statistics (Supporting Information 4). Firstly, an intercept‐only model was tested, which implies no systematic change as a function of time. Second, a linear growth model was tested with the inclusion of a second correlated factor. The latent growth process in this model was captured by an initial level of growth (intercept) and a linear slope. Third, the quadratic growth model was tested, where an intercept, a linear slope and a quadratic slope captured the latent growth process. Next, the piecewise linear growth curve model was tested. The latent growth process here is captured by tying two or more linear pieces at a point on the curve corresponding to an inflection point. Two linear pieces were tied together, and thus, two linear factors were included in the model. After examining the pattern of means and fitting the models with different knots, the optimal knot point was considered to be the second time point. Last, the latent basis model was tested, where the latent growth process was captured by the intercept and a slope with some of its loadings freely estimated. The factor loadings between the first and the last measurement occasion were freely estimated. After identifying the optimal functional form separately for the language, theta power and alpha power LGCMs, two multivariate LGCMs were tested to estimate the simultaneous growth processes for language and theta power and for language and alpha power, examining how the two constructs from each model travelled together through time. Both models were parametarised so that each slope factor was regressed on the across‐domain intercept factor to test the extent to which the starting point on theta or alpha power in part predicted rates of change on language and vice versa. As a final step, the multivariate LGCMs were re‐run, including autism likelihood group, biological sex and maternal education as predictors of the intercept and slope factors of language and theta power and language and alpha power. Although there were no significant group differences in sex, it was included as a covariate in the final models given its known associations with language development and standard practice in developmental research. Controlling for sex helped account for potential individual differences and improve model accuracy. For a detailed description of the analytic procedure, refer to Supporting Information 5.

In addition, supplementary analysis was performed to examine whether the relationship between the intercept of theta power and the slope of language changed depending on the autism likelihood group. The intercept of theta and the slope of language factor scores were extracted from the multivariate LGCM and were included in a regression model, with the slope of language being the dependent variable and the intercept of theta power, autism likelihood group, as well as their interaction as the independent variables.

Missing data were assumed to be missing at random (MAR) and were accommodated using Full Information Maximum Likelihood (FIML) estimation, using all the available information from the data. This approach provides unbiased parameter estimates and is well‐suited for longitudinal data with incomplete observations (Enders and Bandalos 2001). Maximum likelihood with robust standard errors (MLR) was used to correct for multivariate nonnormality of the residuals of the observed scores (Table S3, Figures S4A and S4B), adjusting standard errors and the overall test statistic. All models were run in R (version 4.2.2) using the lavaan package (Rosseel 2012).

## 3 Results

First, univariate LGCMs were run to determine the best‐fitting functional form of growth for language, frontal theta power and frontal alpha power separately. Second, multivariate LGCMs were used to examine how trajectories of EEG power and language related to each other over time. Finally, these models were extended to include covariates (biological sex, maternal education, and autism likelihood group) (see Table S4 for an overview of the statistical models tested).

### 3.1 Univariate LGCMs

For the language domain, the latent basis model was selected over the linear, quadratic and piecewise models, as it best represented the functional form of growth for language, providing an acceptable fit to the data in terms of the comparative fit index (CFI = 0.942), the standardized root mean square residual (SRMR = 0.060) and less so in terms of Chi‐Square (χ^2^(3) = 12.138, *p* = 0.007) and the root mean square error of approximation (RMSEA = 0.138). Refer to Supporting Information 6 and Tables S5 and S6 for the results of model fit statistics and parameter estimates. The estimates of the individual trajectories are shown in Figure S5.

For the frontal relative theta power domain, the piecewise linear growth model with the residual variances across time constrained to equality was selected as the one that best represented the functional form of growth for theta power (χ^2^(4) = 5.881, *p* = 0.208, CFI = 0.994, RMSEA = 0.045 [0.000–0.120], SRMR = 0.022) over the linear, quadratic and latent basis models. The results of model fit statistics and parameter estimates are described in detail in Supporting Information 6 and Tables S7 and S8. The estimates of the individual trajectories are illustrated in Figure S6.

For the frontal relative alpha power domain, the piecewise linear model with the residual variances constrained to equality provided an acceptable fit to the data in terms of CFI (0.942) and SRMR (0.041) and less so in terms of Chi‐Square (χ^2^(4) = 20.584, *p* < 0.001) and RMSEA (0.142). Given that the piecewise model was more parsimonious and resulted in a proper solution with acceptable standalone fit indices, it was selected as the one adequately representing the functional form of growth for alpha power over the other models (see Supporting Information 6 and Tables S9 and S10 for the detailed results of model fit statistics and parameter estimates). The estimates of the alpha power individual trajectories are illustrated in Figure S7.

### 3.2 Multivariate LGCMs

#### 3.2.1 Frontal Relative Theta Power and Language

The final univariate model of theta power was tested simultaneously with the final univariate model of language to examine their relationship at 10 months, whether a change in theta power was related to a change in language, as well as the extent to which theta power at 10 months predicted rates of change in language and vice versa. The Multivariate LGCM of theta power and language fitted the data satisfactorily (χ^2^(17) = 29.984, *p* = 0.026, CFI = 0.965, RMSEA = 0.073 [0.010–0.119], SRMR = 0.048). The intercept of theta power as a predictor of the slope of language was marginally significant (Estimate = –3.961, *SE* = 2.177, *z *= –1.819, *p* = 0.069) with a standardised coefficient of –0.27. The intercept of language did not significantly predict the pre (Estimate = –0.007, *SE* = 0.012, *z* = –0.528, *p* = 0.598) and postknot (Estimate = 0.002, *SE* = 0.002, *z* = 0.794, *p* = 0.427) slopes of theta power. The covariance between the intercepts and slopes of theta power and language was nonsignificant (*ps *> 0.118). Regarding effect size measures, *r*‐squared values showed that the intercept of theta power explained 7% of the variance of the slope of language, while approximately 2% of the variance of the pre and postknot slopes of theta power was explained by the intercept of language. Table S11 provides details of the parameter estimates of the language and theta power Multivariate LGCM.

#### 3.2.2 Frontal Relative Alpha Power and Language

Next, the final univariate model of alpha power was tested simultaneously with the final univariate model of language to examine their relationship at 10 months, whether a change in alpha power was related to a change in language as well as the extent to which alpha power at 10 months predicted rates of change in language and vice versa. The Multivariate LGCM of alpha power and language fitted the data adequately (χ^2^(17) = 35.185, *p* = 0.006, CFI = 0.949, RMSEA = 0.087 [0.035–0.135], SRMR = 0.049). The intercept of alpha power was a nonsignificant predictor of the slope of language (Estimate = 1.512, *SE* = 2.304, *z *= 0.656, *p* = 0.512). The intercept of language did not significantly predict the pre (Estimate = –0.001, *SE* = 0.011, *z* = –0.110, *p* = 0.912) and postknot (Estimate = –0.002, *SE* = 0.002, *z* = –0.802, *p* = 0.423) slopes of alpha power. The covariance between the intercepts and slopes of alpha power and language was nonsignificant (*ps* > 0.180). In terms of effect size measures, *r*‐squared values showed that the intercept of alpha power explained approximately 1% of the variance of the slope of language, while 0% and 1% of the variances of the pre and postknot slopes of theta power, respectively, were explained by the intercept of language. Table S12 provides details of the parameter estimates of the language and alpha power Multivariate LGCM.

### 3.3 Multivariate LGCMs Controlling for the Effects of Sex, Maternal Education and Autism Likelihood Group

As a final step, MLGCMs were re‐run, controlling for the effects of biological sex, maternal education and autism likelihood group. We included autism likelihood group as a covariate in these models as it allowed us to formally test for group differences in both baseline levels and growth trajectories of language and EEG power.

#### 3.3.1 Frontal Relative Theta Power and Language

**FIGURE 1:**
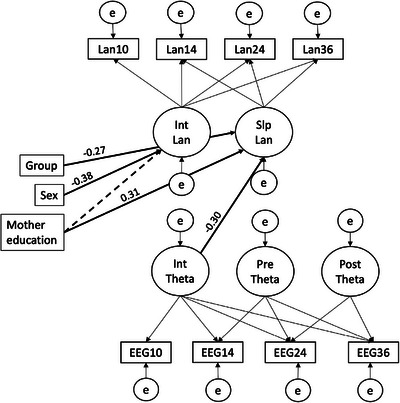
The multivariate latent growth curve model regressed on autism likelihood group, biological sex and maternal education.

The theta power and language Multivariate LGCM fitted the data well (χ^2^(26) = 36.668, *p* = 0.080, CFI = 0.969, RMSEA = 0.058 [0.000–0.099], SRMR = 0.040). With the inclusion of the covariates, the intercept of theta power was a significant predictor of developmental change in language (Estimate = –4.435, *SE* = 2.121, *z* = –2.091, *p* = 0.037) with a standardised coefficient of –0.30, showing that higher theta power at baseline (10 months) predicted less steep slopes in language ability. Further, males showed lower initial scores on language skills than females (Estimate = –0.455, *SE* = 0.115, *z* = –3.953, *p* < 0.001) but did not differ in the rate of change in language scores over time. Maternal education was a marginally significant predictor of language ability at baseline (Estimate = 0.133, *SE* = 0.077, *z* = 1.733, *p* = 0.083) and a highly significant predictor of developmental change in language ability (Estimate = 0.300, *SE* = 0.106, *z* = 2.819, *p* = 0.005). Autism likelihood group was a significant predictor of the slope of language, indicating that infants at EL showed less steep increases in language scores over time compared to infants at TL (Estimate = –0.408, *SE* = 0.168, *z* = –2.422, *p* = 0.015). In terms of effect size measures, *r*‐squared values showed that the proportion of variance explained by the covariates was 2% for the intercept and 29% for the slope of language. Additionally, it was 2% for the intercept and 21%–15% for the pre and postknot slopes of theta power, respectively. No other covariate effects were significantly different from zero. The model is illustrated in Figure 1 and the parameter estimates of the model are provided in Table S13.

First, to test whether the observed differences between the EL and TL group in the slope of language were due to those individuals who went on to an autism diagnosis at 36 months, we re‐ran the model excluding the EL‐autism group (*N* = 12) from the dataset, which did not make a substantial difference to the results. Although the effect slightly decreased, autism likelihood group remained a significant predictor of the slope of language (Estimate = –0.386, *SE* = 0.191, *z* = –2.020, *p* = 0.043). In addition to this analysis, we re‐ran the model including EL‐autism and EL‐no autism groups as separate covariates. The intercept of theta power became a marginally nonsignificant predictor of the slope of language (Estimate = –3.824, *SE* = 2.116, *z* = –1.808, *p* = 0.071). The EL‐autism group had significantly lower scores in language at 10 months than the TL group (Estimate = –0.573, *SE* = 0.173, *z* = –3.310, *p* = 0.001; Supporting Information 7).

**FIGURE 2:**
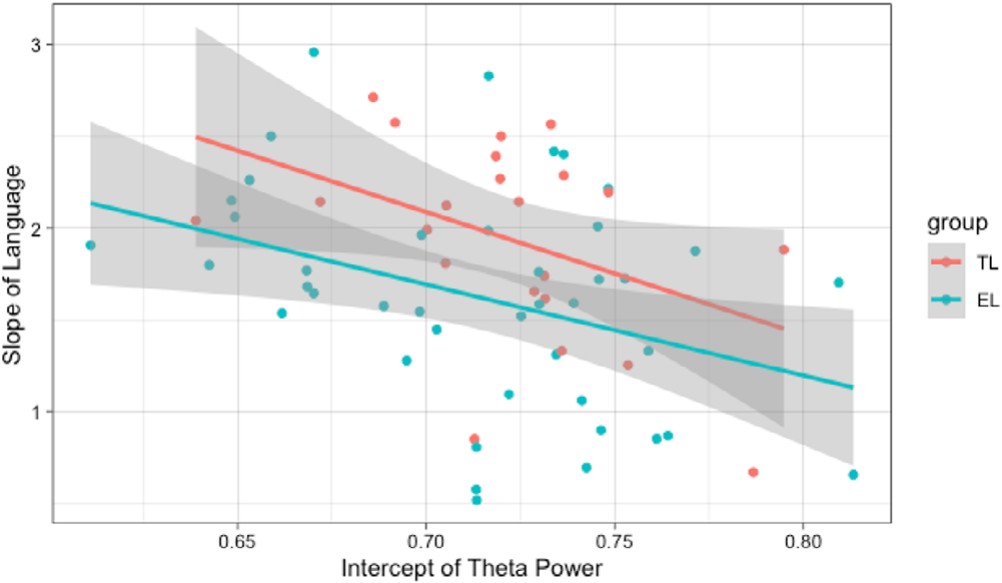
Scatterplot showing theta power factor scores at 10 months as predictor of the slope of language factor scores by autism likelihood group.

Supplementary analysis examining whether the relationship between the intercept of theta power and the slope of language changed depending on autism likelihood group showed the effect of theta power at 10 months on developmental change in language was not dependent on group (*b* = 0.91, *t*(152) = 0.46, *p* = 0.645; Figure 2).

Furthermore, given the identified links between theta power and language, we examined potential condition‐specific effects by analysing theta power to the social and non‐social conditions separately in relation to language. The results revealed that the intercept of theta power responses to the social condition was a significant predictor of developmental change in language (Estimate = –4.090, *SE* = 1.990, *z* = –2.055, *p* = 0.040) with a standardised coefficient of –0.29. In turn, the intercept of theta power to non‐social stimuli was not a significant predictor of developmental change in language (Estimate = –3.531, *SE* = 2.188, *z* = –1.614, *p* = 0.107), indicating that the effect was driven by the social condition (see Supporting Information 8 for details).

To further clarify whether the observed effects were driven by condition differences, we conducted additional analyses examining whether spectral power differences between the social and non‐social conditions were associated with language development. This analysis did not yield results that added to the main conclusions (Supporting Information 9). Furthermore, considering previously reported language‐related lateralisation effects (Lai et al. 2024; Morrel et al. 2023), we conducted a follow‐up analysis to examine whether the identified relationship between theta power and language was driven by lateralisation effects (refer to Supporting Information 10 for a detailed description). The intercept factors did not significantly predict the cross‐domain slope factors (*ps* > 0.1), suggesting that within this sample, individual differences in brain asymmetry were not predictive of language development trajectories and vice versa. We further separated the language variable into expressive and receptive components to examine whether the association with theta power was specific to one aspect of language. The intercept of theta power was a significant negative predictor of the slope of receptive language (Estimate = –6.070, *SE* = 2.075, *z* = –2.925, *p* = 0.003). No significant associations were observed between the intercept and slope factors for expressive language (*ps* > 0.1, Supporting Information 11).

Last, to examine whether these findings were specific to language or reflected general cognitive development, we tested if similar relationships existed with the fine motor MSEL domain (Supporting Information 12). Since this variable had not been included in the main models, we first ran LGCMS to define its optimal functional form of growth. The results showed nonsignificant associations between the intercept of theta power and the fine motor slope factor (Estimate = –4.741, *SE* = 2.532, *z* = –1.872, *p* = 0.061), indicating that the association was more specific to language than general cognitive development in the current sample.

#### 3.3.2 Frontal Relative Alpha Power and Language

**FIGURE 3:**
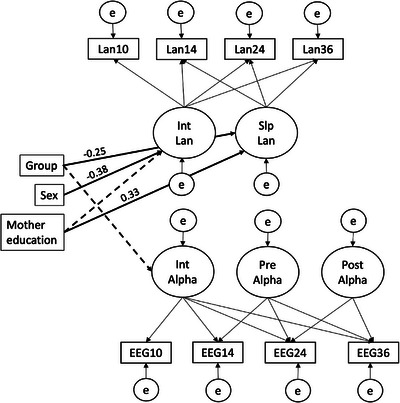
The multivariate latent growth curve model regressed on autism likelihood group, biological sex and maternal education.

The alpha power and language Multivariate LGCM fitted the data adequately (χ^2^(26) = 48.717, *p* = 0.004, CFI = 0.932, RMSEA = 0.087 [0.045–0.126], SRMR = 0.040). As in the unconditional MLCM, the intercept factors did not significantly predict the cross‐domain slope factors. Further, males showed lower initial scores on language skills than females (Estimate = –0.456, *SE* = 0.115, *z* = –3.968, *p* < 0.001), but did not differ in the rate of change in language scores over time. Maternal education was a marginally significant predictor of language ability at baseline (Estimate = 0.133, *SE* = 0.077, *z* = 1.736, *p* = 0.083) and a highly significant predictor of developmental change in language ability (Estimate = 0.310, *SE* = 0.107, *z* = 2.910, *p* = 0.004). Last, the autism likelihood group was a significant predictor of the slope of language, showing that infants at EL showed less steep increases in language scores over time compared to infants at TL (Estimate = –0.381, *SE* = 0.172, *z* = –2.212, *p* = 0.027). In addition, the autism likelihood group was a marginally significant predictor of alpha power at the baseline (Estimate = 0.017, *SE* = 0.009, *z* = 1.781, *p* = 0.075). In terms of effect size measures, *r*‐squared values showed that the proportion of variance explained by the covariates ranged between 19% and 21% for the intercept and slope of language. Additionally, it was near 4% for the intercept and 3%–8% for the pre and postknot slopes of alpha power, respectively. No other covariate effects were significantly different from zero. The model is illustrated in Figure 3 and the parameter estimates of the model are provided in Table S14.

After re‐running the model excluding the EL‐autism group from the dataset, the autism likelihood group became a marginally nonsignificant predictor of the slope of language (Estimate = –0.354, *SE* = 0.197, *z* = –1.801, *p* = 0.072). Furthermore, including EL‐autism and EL‐no autism as separate covariates in the model revealed that both groups significantly differed from the TL group in alpha power at 10 months (EL‐no autism: Estimate = 0.023, *SE* = 0.009, *z* = 2.503, *p* = 0.012; EL‐autism: Estimate = –0.023, *SE* = 0.011, *z* = –2.127, *p* = 0.033).

In addition, we examined potential condition‐specific effects by analysing alpha power to the social and non‐social conditions separately in relation to language. The results remained largely consistent with the results of the main model (Refer to Supporting Information 8 for details). Additional analysis examining associations between alpha power differences between the social and non‐social conditions and language development did not provide further insight beyond the main results (Supporting Information 9).

Furthermore, two additional models were run to investigate possible associations between alpha power and expressive or receptive language. No significant associations were observed between the intercept and slope factors across both models (all *ps* > 0.1; Supporting Information 11).

Last, to address the challenge of disentangling theta versus alpha contributions in the relative power measures, we conducted supplementary analyses examining whether absolute theta and alpha power measures and theta‐alpha difference predicted language development. Neither absolute theta power (Estimate = –0.14, *p* = 0.37) nor absolute alpha power (Estimate = –0.12, *p* = 0.42) showed significant associations with language outcomes. Similarly, theta‐alpha difference scores were not predictive of language change (Estimate = –0.14, *p* = 0.29). This pattern of results combined with the main findings (associations between relative theta and language, but not relative alpha) suggests that theta's relative contribution to the power spectrum is most predictive of language development.

## 4 Discussion

Investigating the developmental progression of infant neural activity and language ability and their association over developmental time is important for a greater understanding of the variability in brain and language development as well as the possible developmental processes linked to later language outcomes in the context of autism. To our knowledge, this is the first study examining longitudinal associations between frontal alpha/theta power and language across 10, 14, 24 and 36 months in a sample of infant siblings with an EL and TL for autism. The current study adds to previous research examining EEG power‐language associations cross‐sectionally or at a single time point in one domain in relation to developmental changes in the other. We aimed to develop a better understanding of the neurodevelopmental processes associated with language skill development in infants with TL and EL for autism at multiple time points. We focused on the theta and alpha frequencies in the frontal region, as these frequencies have previously been associated with language outcomes (Huberty et al. 2023; Levin et al. 2017; Wilkinson et al. 2020). Consistent with our hypotheses, controlling for autism likelihood group, sex, and maternal education, we found that theta power to averaged social and non‐social stimuli at 10 months significantly predicted developmental change in language. This association was driven by the theta power responses to the social condition and was mostly associated with receptive language. This association was not moderated by autism likelihood group. Although we failed to identify group differences in the trajectories of theta and alpha power as per our hypothesis, our results showed that infants at TL and EL differed in the trajectories of language ability. In addition, and in contrast to our predictions, no association was found between alpha power and language development. Developmental change in alpha and theta power was also unrelated to developmental change in language ability. Taken together, the study findings shed light on the neurodevelopmental processes underlying language ability in both typical and atypical development by suggesting a continuity in brain and language associations between the infant sibling groups who only seem to differ behaviourally across time.

### 4.1 Autism Likelihood Group as a Predictor of Language

Controlling for biological sex and maternal education revealed that they were both significant predictors of language ability. This result is in line with previous studies showing a relationship between sex and parental education and language development (Campbell et al. 2003; Chilosi et al. 2023; Wilkinson et al. 2020; Zambrana et al. 2012) and highlights the prominent role that both may play in language ability. Another main finding from the current study confirming our predictions was that controlling for sex and maternal education, the infant groups did not differ in their language scores at baseline, but the EL group showed smaller improvements in language scores between 10 and 36 months than the TL group. Re‐running the models after excluding the EL infants who were later diagnosed with autism showed that the results remained largely the same. However, supplementary analysis adding the EL‐autism and EL‐no autism groups as covariates revealed that the EL‐autism group had lower language scores than the TL group at 10 months. Although the alpha power multivariate LGCM revealed a marginally nonsignificant association between autism likelihood group and language slope after the exclusion, this may have been caused due to a reduction in power. Therefore, the results revealed that this association was not solely driven by EL infants who later received an autism diagnosis, and that the EL‐autism subgroup specifically shows early language differences, already evident at 10 months. This aligns with substantial evidence showing that infants with a familial likelihood of autism exhibit delayed development in their language skills and that differences in behavioural characteristics between the sibling groups become more observable from around the first year of life (Hudry et al. 2014; Jones et al. 2014; Marrus et al. 2018; Messinger et al. 2013; Ozonoff et al. 2011; Szatmari et al. 2016). This result places language ability as a trait symptom shared across infants at EL with common genetic liability and possible neurobiology (Marrus et al. 2018). Furthermore, while the EL and TL infant groups did not differ in EEG power in the main analysis, possibly due to limited variability in both alpha and theta frequency ranges across the whole sample, we did observe significant group differences in alpha power at 10 months. Specifically, infants later diagnosed with autism showed reduced alpha power, while EL‐no autism infants showed increased alpha power relative to TL controls. This early group differentiation aligns with prior findings reporting differences between EL and TL infants in EEG power across different frequency bands, including alpha (Levin et al. 2017; Tierney et al. 2012). However, it is also consistent with more recent cross‐sectional work showing similarities in theta power between EL and TL infants at 14 months (Haartsen et al. 2022). Taken together, the findings suggest (within this sample) that the factors associated with language delay may not be specific to increased likelihood of autism and that group differences in alpha power may emerge early in infancy, but developmental trajectories across groups may converge or remain stable thereafter. It remains possible that, compared to alpha and theta power, other measures of brain function, structure, or social/non‐social processing might be more informative or would be able to capture more accurately the potential neurodevelopmental mechanisms underlying language difficulties in infants at EL for autism (e.g., measures including cross‐frequency phase‐amplitude coupling have also been linked to language in neurotypical populations, Attaheri et al. 2022; Lizarazu et al. 2019). Future research could entail multiple possible brain biomarkers in association with language development in the context of autism.

### 4.2 Linking EEG Spectral Power and Language

The association between 10‐month frontal theta power intercept and the degree of change in language observed in the current study is similar to prior research showing increased theta power at 6 months or power changes from 6 to 12 months during the watching of dynamic semi‐naturalistic videos being associated with 24‐month language or verbal skills in infants with EL (Jones et al. 2020; Wilkinson et al. 2020). It also coincides with previous research showing links between increased EEG power spectral density in the theta range during infant‐directed speech in the first year of life and poorer language outcomes at 24 months in typically developing infants (Attaheri et al. 2022). Interestingly, in the current study theta power was significantly associated with the slope of language while no concurrent (at 10 months) associations were observed. The results thus show that theta power may not be a correlate of early communication skills (at least for this study) but may represent a critical neural mechanism explaining individual differences in the developmental trajectories of language ability. Indeed, during language processing, modulations in theta oscillations are observed and appear to become tuned to the syllabic rhythm of speech (i.e., infant‐directed speech or nursery rhymes), which is essential for language acquisition (Attaheri et al. 2022; Giraud and Poeppel 2012; Goswami 2019; Leong et al. 2017). Further, theta oscillatory activity has been found to increase during infant‐directed speech and toy exploration (Orekhova et al. 2006). It has been associated with cognitive functions such as reward anticipation, active learning, encoding of new information and attention allocation (Begus and Bonawitz 2020; Gruber et al. 2013; Sauseng et al. 2007). Thus, these processes may have been elicited upon the presentation of social and non‐social videos. However, this possibility contradicts our findings for negligible associations between developmental change of language and theta power. Although we cannot be certain of the specificity of the mechanisms underlying theta power activity, it likely reflects a joint operation of these processes early on, shaping infants’ capacity for language acquisition over time, and that, a change in these processes possibly related to theta power activity may not necessarily align with a developmental change in language, especially when more factors and environmental inputs are involved as infants develop. Early on, this may represent a ‘canalisation’ process, where the interaction of multiple systems in response to social and non‐social stimuli affecting brain adaptation and plasticity plays a role in developmental trajectories (Elsabbagh and Johnson 2010). Thus, the results suggest that theta power might represent a candidate biomarker of later language ability. This can have important implications for designing early effective therapeutic interventions (possibly focusing on the brain mechanisms that shape infants’ language ability) and improving the prognosis of poor language outcomes.

The absence of frontal alpha power and language associations also contradicts previous findings (Huberty et al. 2023; Levin et al. 2017) that have reported associations between the two measures, one showing concurrent associations at 6 months and the other showing 3‐month alpha power linked to 12‐month language ability. The discrepancy in findings might be due to the study design used. For instance, both studies distinguished between expressive and receptive language and examined associations at different time points. Alpha power was associated with expressive language in these studies. Thus, averaging the two MSEL scales in the current study may have obscured possible effects specific to expressive or receptive language. However, supplementary analyses addressing this showed no significant associations between alpha power and either expressive or receptive language, suggesting that the null finding is unlikely to be due to score averaging. On the other hand, the result may indicate age‐related changes in the relationships, such that while these are shown earlier (i.e., at 6 months), they disappear with age, where other possible factors (i.e., environmental) may come into play. Examining alpha power and language developmental trajectories within the first year could possibly help to delineate this. In contrast, theta power was significantly associated with receptive, but not expressive, language, indicating that the observed effects may be primarily driven by receptive language abilities. Importantly, this aligns well with the nature of the task, during which children were passively viewing videos, a context that engages receptive rather than expressive language processes. This increases our confidence in the findings and supports the interpretation that receptive language skills may be more closely tied to oscillatory activity in this context. In addition, considering the bidirectional influences between brain development and behaviour, we hypothesised that changes in EEG spectral power would be related to changes in language. A possible reason for the absence of this association could be that changes in language and EEG power might be confounded by other factors (environmental or biological) influencing the variation in both domains across development.

Furthermore, while infants at EL seemed to have lower scores in language compared to infants at TL, the association between baseline frontal theta power and the slope of language was not moderated by the autism likelihood group. This suggests that the association between spectral power and language appears to be similar in infants with and without an EL for autism. This may have been due to limited statistical power as a result of testing interaction terms. However, it confirms our hypothesis and agrees with prior research showing EEG power and language associations in both neurotypical populations and those with raised likelihood for neurodevelopmental conditions (Benasich et al. 2008; Brito et al. 2016; Cantiani et al. 2019; Gou et al. 2011; Huberty et al. 2023; Jones et al. 2020; Levin et al. 2017; Lum et al. 2022; Pierce et al. 2019; Wilkinson et al. 2020). Thus, the association between theta power and language ability might not be specific to the TL group but instead generally related to language ability.

### 4.3 Limitations

The study has limitations. Although growth curve models within a structural equation modelling framework allow one to test hypotheses based on theory and examine different relationships within a more parsimonious model, we acknowledge that we did not correct for multiple comparisons in our analyses. Thus, replication of the current findings is necessary to make strong inferences from the results. In addition, the study's sample size was reasonable, but replication with a larger and more balanced number of EL and TL infants (possibly through multi‐site collaborations) could increase statistical power to observe more correlational effects. Furthermore, the language assessments at 10–14 and 24–36 months were conducted at different sites. The increase in average language skills between these time points was attributed to this site difference. Including ‘site’ as a covariate in our models caused convergence issues, therefore it was excluded. However, this does not undermine our main findings, as our focus was on modelling individual differences rather than group means. In addition, the use of relative EEG power may not fully account for developmental variability in peak frequency. However, this approach was deemed the most appropriate for the current sample, as many children in this age range do not exhibit clear or stable spectral peaks, and using peak‐based methods could introduce bias or limit sample inclusion due to missing or unreliable estimates. Last, although shifts in dominant peak frequency from theta to alpha during early development may underlie some of the observed spectral power changes, many infants do not exhibit a clear spectral peak, limiting our ability to assess whether peak frequency was associated with language development, a known challenge in infant EEG research. Moreover, it is difficult to completely disentangle the specific contributions of the theta versus alpha power measures in relation to language. Our pattern of findings showing significant associations for relative theta but not relative alpha and language, combined with null results for absolute power measures suggest relative theta specificity and is consistent with a growing body of literature demonstrating the role of theta in language development (Attaheri et al. 2022; Beese et al. 2017; Jones et al. 2020; Lum et al. 2022; Ortiz‐Mantilla et al. 2019). However, we cannot fully rule out indirect influences of alpha power on our theta‐related findings.

## 5 Conclusion

Overall, the current study has provided insight into the developmental trajectories of EEG power and language ability in infants with and without an EL for autism and into the possible neurodevelopmental mechanisms linked to language development, which can have implications for early intervention. The findings show that frontal theta power significantly predicts language developmental change for EL and TL infants. This reflects a neurodevelopmental process linked to language ability that is similar for both EL and TL infants. It suggests that theta power is a potential predictive biomarker of language ability in infancy. The findings also suggest differences between EL and TL groups in the developmental language change, which provides further insight into language delay in infants at EL as a potential endophenotype of autism. The study also provides evidence for an absence in the association between frontal alpha power and language and shows that EEG power is not moderated by autism likelihood group. Last, the study results support incorporating additional brain function measures by future research for a more holistic view of how developmental change in EEG spectral power relates to language development in EL and TL infants. Examining the genetic or environmental effects on brain measures related to language development will also help inform our understanding of the factors associated with language outcomes and facilitate their early prediction.

## Author Contributions

**Eirini Papageorgopoulou**: conceptualization, visualization, methodology, formal analysis, data curation, writing – original draft. **Jannath Begum Ali**: investigation, data duration, resources, writing – review and editing. **Greg Pasco**: investigation, data curation, writing – review and editing. **Amy Goodwin**: investigation, data curation, writing – review and editing. **Luke Mason**: data curation, writing – review and editing. **Mark H Johnson**: project administration, funding acquisition, writing – review and editing. **Tony Charman**: project administration, funding acquisition, writing – review and editing. **Kalinka Timmer**: supervision, conceptualization, writing – review and editing. **Emily JH Jones**: supervision, conceptualization, writing – review and editing.

## Policy on Using ChatGPT and Similar AI Tools

The authors acknowledge the use of ChatGPT and Claude default versions, accessed between August 2025 and February 2026, during the revision process of the manuscript to refine and rephrase already structured sentences or parts of text for clarity and flow (AI tools were not used during the initial submission or to create the original manuscript). All AI‐assisted text was reviewed and revised by the authors to ensure accuracy and clarity of meaning.

## Funding

This work was supported by the Horizon 2020 Marie Skłodowska‐Curie Action (MSCA) Innovative Training Network (ITN)—European Training Network (ETN), grant number: 814302 – SAPIENS). This research was also supported by awards from the Medical Research Council (MR/K021389/1; M.H.J., T.C.) and (MR/T003057/1; E.J.H.J., M.H.J., T.C.), MQ (MQ14PP_83, M.H.J., E.J.H.J., T.C.). Further, the work was supported by the EU‐AIMS and AIMS‐2‐TRIALS programmes funded by the Innovative Medicines Initiative (IMI) Joint Undertaking Grant Nos. 115300 (M.H.J., T.C.) and No. 777394 (M.H.J., E.J.H.J. and T.C.; European Union's FP7 and Horizon 2020, respectively). This Joint Undertaking receives support from the European Union's Horizon 2020 research and innovation programme, with in‐kind contributions from the European Federation of Pharmaceutical Industries and Associations (EFPIA) companies and funding from Autism Speaks, Autistica and SFARI.

## Disclaimer

The funders had no role in the design of the study; in the collection, analyses or interpretation of data; in the writing of the manuscript, or in the decision to publish the results. Any views expressed are those of the author(s) and not necessarily those of the funders.

## Ethics Statement

Ethical approval was granted by the National Research Ethics Service (13/LO/0751 and 08/H0718/76) and the Research Ethics Committee of the Department of Psychological Sciences, Birkbeck, University of London.

## Conflicts of Interest

T.C. has served as a paid consultant to F. Hoffmann‐La Roche Ltd. and received royalties from Sage Publications and Guildford Publications.

## Supporting information




**Supporting file**: desc70194‐sup‐0001‐SuppMat.docx

## Data Availability

The datasets used in this study are stored in the STAARS Network Data Repository. The conditions of our ethics approval do not allow for public archiving of the pseudonymised study^1^ data. Data cannot be fully anonymized due to the nature of combined sources of information, such as sociodemographic and clinical outcome measures, making it possible to attribute data to specific individuals, and hence, falling under personal information, the release of which would not be compliant with GDPR guidelines unless additional participant consent forms were completed. Our data sharing procedures were created in consultation with stakeholders (Begum‐Ali et al. 2024). To access the data, interested readers should contact the STAARS network coordinator(s) at my and Emily's emails. Access may be granted upon completion of a successful Project Affiliation Form via the STAARS network (www.staars.org). Additionally, requestors must complete and sign a data sharing agreement to ensure data is stored securely. Approved projects would need to adhere to the network's policies on Ethics, Data Sharing, Authorship and Publication. Please refer to the data sharing policies available at www.staars.org for further details on the data access process and requirements. No part of the study procedures or analysis plans was preregistered prior to the research being conducted.
