# Supplementary material for: Theta Power at 10 Months of Age Predicts Developmental Change in Language in Infants With and Without an Elevated Likelihood for Autism
Source: Dev Sci. 2026 Apr 13;29:e70194. doi: 10.1111/desc.70194 (PMC13073434; doi:10.1111/desc.70194)
Supplement: Supplementary file 1 — Supporting file: desc70194‐sup‐0001‐SuppMat.docx [file DESC-29-e70194-s001.docx]

**Theta Power at 10 Months of Age Predicts Developmental Change in Language in Infants With and Without an Elevated Likelihood for Autism Spectrum Disorder -**

**Supporting Information**

**Supporting Information 1. Number of trials: Group comparisons**

We compared the number of valid trials across likelihood (TL vs. EL) and outcome groups (TL, EL-no-autism, EL-autism) at each age point (10, 14, 24, and 36 months) and for both the social and non-social conditions (**Table S1**). There were **nonsignificant differences** in the number of trials between TL and EL groups at any age or condition (all ps > .52), nor between the three outcome groups (all ps > .29). Mean trial counts ranged from approximately 81 to 144 trials per condition, well above the minimum threshold of 10 trials used for inclusion.

**Supporting Information 2. Experiment procedure**

Procedures were put in place in case the infant displayed negative affect avoidance behaviour during the experiment, as described in Begum-Ali et al. (2022). Infant fussiness can lead to poor quality or missing data. To maximise data collection and participant comfort, a number of steps were followed in a hierarchical manner. Firstly, the parents were asked to ensure their infants were comfortable (warm, fed, changed and seated comfortably) before the experiment. During the experiment, if infants were fussy, researchers played non-social ‘attention-getters’ through speakers. If this failed to work, the examiner spoke to the infant (this information was inserted into the datafile through manual coding). If this did not work, the parent was asked to try cuddling, holding hands, giving the infant something boring to hold (i.e., a plastic teething ring), give the infant a dummy or a snack. If fussiness continued, the dyad was instructed to take a break and the experiment was resumed upon agreement with the parent and after ensuring that the infant was calm (Jones et al., 2019a).

**Supporting information 3. Mullen Scales of Early Learning**

The MSEL was administered by trained researchers in the STAARS team (Begum-Ali et al., 2022). To allow for the greatest level of replicability and consistency across examiners, we used strict guidelines about how the Mullen (Mullen, 1995) should be administered and marked. To this end, our guidelines for Mullen scoring include only behaviours that are captured on camera (so can be confirmed by a second/third researcher if necessary) within the Mullen session. To further ensure the fidelity of the scoring, a second fully trained researcher watches the administration in real time (via a video feed) and consensus discussions take place after the testing session. These strict administration and scoring guidelines (although those recommended in the Mullen manual) may not be those applied more broadly in the field, and thus may account for relatively poorer performance in this cohort at infant timepoints relative to US norms (see Jones et al., 2019).

**Supporting Information 4. Model Fit**

The following fit indices were used to evaluate the standalone fit of the models: the Chi-Square (χ^2^) statistic (though considering that this statistic is sensitive to sample size and assumes multivariate normality, and could reject a properly specified model (Bentler & Bonett, 1980; McIntosh, 2007)), the comparative fit index (CFI), the root mean square error of approximation (RMSEA), and the standardized root mean square residual (SRMR). Although debatable (Marsh et al., 2004), Chi-Square p-value > 0.05, CFI > 0.95 or > 0.90 (acceptable fit), RMSEA < 0.06 or < 0.07 (or less conservatively, < 0.08) and SRMR < 0.08 indicate acceptable fit (Hu & Bentler, 1999; Schermelleh-Engel et al., 2003; Steiger, 2007).

The relative fit between the LGCMs was assessed through the Bayesian Information Criterion (BIC). In small or moderate sample sizes, the BIC outperforms other model selection criteria (i.e., Akaike information criterion (AIC)) in terms of the selection of the correct model structure (Neath & Cavanaugh, 2012). Lower BIC values indicate a better fit. A difference between the BIC values from 2-6 is considered ‘positive’ evidence, 6-10 ‘strong’, while a difference of > 10 is considered ‘very strong’ evidence (Neath & Cavanaugh, 2012). Where the BIC values were < 2, the AIC was considered. Lower values indicated a better fit and a difference between the AIC values of more than two was indicative of ‘strong’ evidence (Burnham & Anderson, 2004).

**Supporting Information 5. Analytic Procedure**

Before examining the links between language skills and theta/alpha spectral power over developmental time, a series of LGCMs were run to test for the optimal functional form of growth separately for language, theta power and alpha power. Firstly, an intercept-only model was tested, which implies no systematic change as a function of time. Only the intercept factor was specified, fixing all factor loadings to one. The factor mean, variance, and the time-specific residual variance were estimated.

Second, a linear growth model was tested with the inclusion of a second correlated factor. The latent growth process in this model was captured by an initial level of growth (intercept) and a linear slope. The means and variances of the latent factors, the covariances between them, as well as the time-specific residual variances were estimated (the model was also tested with residual variances constrained to equality on some occasions – see results section in the article for details). The intercept factor loadings were fixed to one, as in the intercept-only model. The factor loadings of the linear slope were initially set so that they represented the time elapsed between measurement occasions, approximately in 6-month increments: age 10 was set to 0 (no time elapsed), age 14 was set to 1, age 24 was set to 3 and age 36 was set to 5. The choice to code time in this way was considered to better capture the data because a) individual participant age varied in each measurement occasion, b) previous attempts precisely set the factor loadings to represent the course of time in months (i.e., age 10 would be set to 0, age 14 to 4 (14 – 10 = 4), age 24 to 14, age 36 to 26) and years (0, 0.34, 1.17, 2.17) based on the distance from the baseline led to model fit and estimation issues.

Third, the quadratic growth model was tested, where an intercept, a linear slope and a quadratic slope captured the latent growth process. The means and variances of the three latent factors and the covariances between them were estimated. The time-specific residual variances were also estimated (the model was also tested with residual variances constrained to equality on some occasions – see results section for details). The factor loadings of the intercept and linear slope remained the same, while the quadratic slope factor loadings were the squared values of the linear slope factor loadings.

Next, the piecewise linear growth curve model was tested. The latent growth process here is captured by tying two or more linear pieces at a point on the curve that corresponds to an inflection point. Two linear pieces were tied together, and thus, two linear factors were included in the model. After examining the pattern of means and fitting the models with different knots, the optimal knot point was considered to be the second time point. The means and variances of the three latent factors as well as their covariances, were estimated. The residual variances were constrained to equality (see results section for details). Again, the intercept factor loadings were fixed to one. The loadings of the two linear slopes were fixed in a way that defined the two linear pieces that made up the trajectory: the first piece of the linear slope (first latent factor-preknot point) was defined from 10 to 14 months and stopped incrementing from 14 to 36 months. The second piece of the linear slope (second latent factor-postknot point) was fixed to zero at the preknot point and incremented after the 14-month time point up to 36 months.

Last, the latent basis model was tested, where the latent growth process was captured by the intercept and a slope with some of its loadings freely estimated. The factor loadings between the first and the last measurement occasion were freely estimated. The means and variances of the latent factors, the covariances between them as well as the time-specific residual variances were estimated. The mean of the slope factor represented the total predicted amount of change from the first to the last measurement occasion. The estimated factor loading represented the proportion of total change up until the associated time point. The factor loadings of the slope factor were fixed to zero at the age of 10 months, were freely estimated at 14 and 24 months and fixed to one at the age of 36 months.

After identifying the optimal functional form separately for the language, theta power and alpha power LGCMs, two multivariate LGCMs were tested to estimate the simultaneous growth processes for language and theta power and for language and alpha power, examining how the two constructs from each model travelled together through time. Both models were parametarised so that each slope factor was regressed on the across-domain intercept factor to test the extent to which the starting point on theta or alpha power in part predicted rates of change on language and vice versa.

Finally, the multivariate LGCMs were re-run, including autism likelihood group, biological sex and maternal education as predictors of the intercept and slope factors of language and theta power as well as language and alpha power.

**Supporting Information 6. Univariate LGCMs**

***Language***

**Table S4** provides the results of model fit of the language LGCM. The intercept-only model fitted the data poorly. The linear growth model also provided a poor fit to the data, with the model resulting in a negative variance estimate for the ‘language’ observed variable at 36 months. Fixing the variance to zero did not improve model fit. The linear model was expanded with the inclusion of a quadratic factor, which fitted the data extremely poorly and resulted in negative variance estimates for the language observed variable, intercept, linear and quadratic slope factors. This indicated that the quadratic model was not appropriate for the data. Fixing the variances to zero resulted in model estimation failure. The piecewise linear model resulted in an improper solution, with the covariance matrix of latent variables being positive definite and with negative variance estimates. Fixing the variances to zero resulted in model estimation failure. No further adjustments were made. The latent basis model provided an acceptable fit to the data in terms of the comparative fit index (CFI = 0.942), the standardized root mean square residual (SRMR = 0.060) and less so in terms of Chi-Square (χ^2^(3) = 12.138, *p* = 0.007) and the root mean square error of approximation (RMSEA = 0.138). Owing to its acceptable standalone fit, the latent basis model was selected as it best represented the functional form of growth for language. While the RMSEA value exceeded conventional thresholds for acceptable fit (RMSEA = 0.138), it is worth noting that RMSEA can be overly sensitive in models with low degrees of freedom and can be affected by the sample size, leading to inflated estimates of poor fit (Kenny et al., 2015). Therefore, other fit indices were selected (CFI, SRMR) indicating acceptable model fit.

The parameter estimates of the language latent basis model are shown in **Table S6**. The results of the model showed that 14% of the total growth in language occurred in the first 4 months, while 84% of growth occurred between 10 and 24 months. Calculating the relative rate of change it was shown that between 10 and 14 months, the relative rate of change was -0.139 / 4 months · 100 = -3.48%. Between 14 and 24 months, the relative rate of change was [0.835 – (-0.139)] / 10 months · 100 = 9.74%, while between 24 and 36 months it was 1.38%. Thus, the results suggested that the most rapid relative rate of change in language scores occurred between 14 and 24 months, followed by a subsequent decline, with the smallest relative rate of change occurring between 24 and 36 months. The average baseline score (intercept) in language was 36.640 (*SE* = 0.722, *z* = 50.755, *p* <0.001). The average total change in language across developmental time was 17.669 (*SE* = 1.019, *z* = 17.348, *p* <0.001). Thus, the average rate of change in language score between 10 and 14 months was (-3.48% · 17.669) = -0.61 per month, 1.72 between 14 and 24 months and 0.24 between 24 and 36 months. The was a significant variability at the intercept (Estimate = 35.547, *SE* = 6.916, *z* = 5.140, *p* <0.001) as well as at the slope, though to a greater extent (Estimate = 56.921, *SE* = 12.889, *z* = 4.416, *p* < 0.001). The covariance between the latent factors was non-significant (Estimate = 3.882, *SE* = 7.571, *z* = 0.513, *p* = 0.608), indicating that individual differences in language at 10 months were unrelated to individual differences in the changes in language from 10 to 36 months. **Figure S4** illustrates the estimates of the individual trajectories of language.

***Frontal Theta Power***

**Table S7** provides the results of model fit of the theta power LGCM. The intercept-only model provided a poor fit to the data. Expanding the model to include a linear slope factor also resulted in a poor fit to the data across most fit indices. Given that the model residual variances ranged from 0.000-0.002, the model was also run with the residuals constrained to equality across time. This more parsimonious model again resulted in a decrement in model fit. No further adjustments were made. The quadratic growth model resulted in a perfect fit but also in an improper solution, with a negative variance of the quadratic factor and the variance-covariance matrix of the estimated parameters appearing to be non-positive definite. Fixing the variance to zero led to an improper solution. In addition, constraining the residual variances to equality led to failure in model convergence. Considering this and given that the fit indices possibly indicated an overfitted model, the quadratic growth model was not accepted. The piecewise linear model resulted in model identification issues and in a negative variance. Constraining the residual variances across time to equality provided a good fit to the data (χ^2^(4) = 5.881, *p* = 0.208, CFI = 0.994, RMSEA = 0.045 [0.000-0.120], SRMR = 0.022). The latent basis model provided a perfect fit to the data, possibly indicating an overfitted model. In the interest of parsimony and to aid interpretation, the piecewise linear growth model was selected as the one that best represented the functional form of growth for theta power.

The parameter estimates of the theta power piecewise linear LGCM are shown in **Table S8**. The results of the model showed that the average theta power at baseline was 0.719 (*SE* = 0.005, *z* = 136.19, *p* < 0.001). The mean preknot slope was -0.035, indicating that the average trajectory of theta power decreased up to 14 months (*SE* = 0.005, *z* = -6.500, *p* < 0.001). The mean postknot slope was -0.011 showing that theta power continued to decrease up to 36 months (*SE* = 0.001, *z* = -9.046, *p* < 0.001). The variability at the intercept was small but significantly different from zero (Estimate = 0.003, *SE* = 0.001, *z* = 4.862, *p* < 0.001), and was nonsignificant at the pre (Estimate = 0.001, *SE* = 0.001, *z* = 0.815, *p* = 0.415), and postknot slopes (Estimate = 0.000, *SE* = 0.000, *z* = 1.497, *p* = 0.134). The intercept-preknot, intercept-postknot and preknot-postknot slope covariances were nonsignificant (Intercept-preknot: estimate = 0.000, *SE* = 0.000, *z* = -0.626, *p* = 0.531; intercept-postknot: estimate = 0.000, *SE* = 0.000, *z* = -1.608, *p* = 0.108; preknot-postknot: estimate = 0.000, *SE* = 0.000, *z* = -0.285, *p* = 0.776). **Figure S6** illustrates the estimates of the individual trajectories of theta power.

***Frontal Alpha Power***

**Table S9** provides the results of model fit of the alpha power LGCM. The intercept-only model provided a poor fit to the data, as expected. The linear growth model also fitted the data poorly. Given that the model residual variances ranged from 0.000-0.002, the model was also run with the residuals constrained to equality across time. Constraining the residual variances to equality did not improve the fit of the model. No further adjustments were made. The quadratic model provided an acceptable fit to the data (except RMSEA) but resulted in the variance-covariance matrix of the estimated parameters appearing to be non-positive definite. Constraining the residual variances across time to equality resulted in a failure of model convergence. No further adjustments were made. The piecewise linear model resulted in model identification issues and negative variance estimates. Constraining the residual variances to equality led to a proper solution: the model provided an acceptable fit to the data in terms of CFI (0.942) and SRMR (0.041) and less so in terms of Chi-Square (χ^2^(4) = 20.584, *p* < 0.001) and RMSEA (0.142). However, as noted earlier, RMSEA can overestimate poor model fit when the model has few degrees of freedom and is sensitive to sample size, which may lead to misleading indications of model misfit. The latent basis model resulted in a negative variance estimate for the alpha power observed variable at 10 months. Fixing the variance to zero provided a perfect fit to the data (χ^2^(4) = 2.302, p = 0.680, CFI = 1.000, RMSEA = 0.000 [0.000-0.155], SRMR = 0.037), though possibly indicating an overfitted model. To aid interpretation and given that the piecewise model was more parsimonious and resulted in a proper solution with acceptable standalone fit indices, it was selected as the one adequately representing the functional form of growth for alpha power.

The parameter estimates of the alpha power piecewise linear LGCM are shown in **Table S10**. The results of the model showed that the average alpha power at baseline was 0.157 (*SE* = 0.004, *z* = 35.932, *p* < 0.001). The mean preknot slope was 0.027, indicating that the average trajectory of alpha power increased up to 14 months (*SE* = 0.005, *z* = 5.259, *p* < 0.001). The mean postknot slope was nonsignificant, indicating no increase or decrease from 14 months up to 36 months (Estimate = 0.001, *SE* = 0.001, *z* = 1.047, *p* = 0.295). The variability at the intercept, pre and postknot slopes was small but significantly different from zero (Intercept: estimate = 0.002, *SE* = 0.000, *z* = 4.759, *p* < 0.001; preknot slope: estimate = 0.002, SE = 0.001, *z* = 2.283, *p* = 0.022; postknot slope: estimate = 0.000, SE = 0.000, *z* = 2.570, *p* = 0.010). The intercept-preknot, intercept-postknot and preknot-postknot slope covariances were nonsignificant (Intercept-preknot: estimate = -0.001, *SE* = 0.001, *z* = -1.591, *p* = 0.112; intercept-postknot: estimate = -0.001, *SE* = 0.000, *z* = -1.617, *p* = 0.106; preknot-postknot: estimate = 0.000, *SE* = 0.000, *z* = -1.049, *p* = 0.294). **Figure S7** illustrates the estimates of the individual trajectories of alpha power.

**Supporting Information 7. Examining Group Differences in Neural Oscillatory Activity and Language Development**

We investigated group-level effects by including both EL-no autism and EL-autism groups as separate dummy-coded variables in the main models. This allowed for direct comparison of language and EEG power trajectories across all three groups (TL, EL-no autism, EL-autism). The model fitted the data well (χ^2^(29) = 41.544, p = 0.062, CFI = 0.964, RMSEA = 0.060 [0.000-0.099], SRMR = 0.043). The intercept of theta power became a marginally nonsignificant predictor of the slope of language (Estimate = -3.824, *SE* = 2.116, *z* = -1.808, *p* = 0.071). Compared to the TL group, the EL-autism group had significantly lower scores in language at 10 months (Estimate = -0.573, *SE* = 0.173, *z* = -3.310, *p* = 0.001). The rest of the associations between the groups and language/theta power were nonsignificant (*ps* > 0.05).

The alpha power model fitted the data adequately (χ^2^(29) = 51.418, p = 0.006, CFI = 0.934, RMSEA = 0.082 [0.034-0.105], SRMR = 0.041). Compared to the TL group, EL-autism group had significantly lower scores in language at 10 months (Estimate = -0.567, *SE* = 0.173, *z* = -3.272, *p* = 0.001). In addition, compared to the TL group, both EL groups significantly differed in alpha power at 10 months (EL-no autism: Estimate = 0.023, *SE* = 0.009, *z* = -2.503, *p* = 0.012; EL-autism: Estimate = -0.023, *SE* = 0.011, *z* = -2.127, *p* = 0.033). No group differences were found in the slopes of language and alpha power.

Overall, these results suggest that while group status accounts for some variability in oscillatory activity and language, associations between early theta power and language development remain at least partially independent of diagnostic group.

**Supporting Information 8. Frontal Theta and Alpha Power Responses to Social and Non-social Conditions and Language**

To investigate potential condition-specific effects in the association between EEG power and language, supplementary analysis was conducted in which theta and alpha power was analysed separately for the social and non-social conditions in relation to language development.

Initial run of the theta power to the social condition and language multivariate LGCM led to model estimation issues. Follow-up steps to address estimation warnings such as re-scaling the theta power latent factors (due to large differences in the scaling of the EEG and language variables) and fixing the negative observed variances to zero did not improve model stability. Subsequently, to enable a better balance between model complexity and sample size, we tested a simpler model by removing biological sex, as we considered it a less critical covariate given that sex did not differ significantly between the TL and EL groups. The model fitted the data well (χ^2^(23) = 30.666, p = 0.131, CFI = 0.975, RMSEA = 0.055 [0.000-0.102], SRMR = 0.042). The results remained broadly consistent with the results of the main model. The intercept of theta power was a significant predictor of developmental change in language (Estimate = -4.090, *SE* = 1.990, *z* = -2.055, *p* = 0.040) with a standardised coefficient of -0.29. The intercept of language did not significantly predict the pre (Estimate = -0.019, *SE* = 0.017, *z* = -1.148, *p* = 0.251) and postknot (Estimate = 0.004, *SE* = 0.003, *z* = 1.241, *p* = 0.215) slopes of theta power. Males showed lower initial scores on language skills than females (Estimate = -0.446, *SE* = 0.112, *z* = -3.970, *p* < 0.001) but did not differ in the rate of change in language scores over time. Maternal education was a significant predictor of language ability at baseline (Estimate = 0.150, *SE* = 0.072, *z* = 2.071, *p* = 0.038) and across time (Estimate = 0.363, *SE* = 0.109, *z* = 3.335, *p* = 0.001). There was a significant negative covariance between the intercept and postknot slope of theta power, meaning that greater theta power at 10 months was related to lower rate of change in theta power at 14-36 months (Estimate = -0.000, *SE* = 0.000, *z* = -2.815, *p* = 0.005). Regarding effect size measures, r-squared values showed that the proportion of variance explained by the covariates was 18% for the intercept and 23% for the slope of language. Additionally, it was 0.8% for the intercept and 23% and 8% for the pre and postknot slopes of theta power, respectively. No other covariate effects were significantly different from zero.

The theta power to non-social stimuli and language multivariate LGCM fitted the data well (χ^2^(26) = 34.768, p = 0.117, CFI = 0.979, RMSEA = 0.047 [0.000-0.087], SRMR = 0.042). The intercept of theta power was not a significant predictor of developmental change in language (Estimate = -3.531, *SE* = 2.188, *z* = -1.614, *p* = 0.107). Similarly, no significant associations were found between the intercept of language and the theta power preknot (Estimate = -0.014, *SE* = 0.015, *z* = -0.938, *p* = 0.348) and postknot slopes (Estimate = 0.005, *SE* = 0.003, *z* = 1.448, *p* = 0.148). Further, males showed lower initial scores on language skills than females (Estimate = -0.456, *SE* = 0.114, *z* = -3.989, *p* < 0.001) but did not show any associations in the rate of change in language scores over time. Maternal education was a highly significant predictor of developmental change in language ability (Estimate = 0.315, *SE* = 0.111, *z* = 2.831, *p* = 0.005). Autism likelihood group was a significant predictor of the slope of language, with EL infants showing less steep increases in language scores over time compared to infants at TL (Estimate = -0.382, *SE* = 0.178, *z* = -2.145, *p* = 0.032). In terms of effect size measures, r-squared values showed that the proportion of variance explained by the covariates was 20% for the intercept and 24% for the slope of language. Additionally, it was 3% for the intercept and 20% for the pre and postknot slopes of theta power. No other covariate effects were significantly different from zero.

We repeated this analysis for alpha power to examine potential condition-specific effects that may have been obscured by collapsing across conditions in the main analysis. The alpha power to social stimuli and language multivariate LGCM fitted the data adequately (χ^2^(26) = 43.012, p = 0.019, CFI = 0.949, RMSEA = 0.065 [0.000-0.087], SRMR = 0.041). As in the main model, the intercept factors did not significantly predict the cross-domain slope factors (all *ps* > 0.1) and males showed lower initial scores on language skills than females (Estimate = -0.459, *SE* = 0.114, *z* = -4.043, *p* < 0.001) but did not differ in the rate of change in language scores over time. Maternal education remained a marginally significant predictor of language ability at baseline (Estimate = 0.133, *SE* = 0.073, *z* = 1.811, *p* = 0.070) and a highly significant predictor of developmental change in language ability (Estimate = 0.330, *SE* = 0.112, *z* = 2.935, *p* = 0.003). Last, the autism likelihood group became a marginally nonsignificant predictor of the slope of language (Estimate = -0.353, *SE* = 0.182, *z* = -1.938, *p* = 0.053). In addition, the autism likelihood group became a significant predictor of alpha power at the baseline (Estimate = 0.019, *SE* = 0.009, *z* = 2.132, *p* = 0.033), showing that the EL group initially showed higher alpha power to social stimuli than the TL group. There was a significant negative covariance between the intercept and postknot slope of alpha power (Estimate = 0.000, *SE* = 0.000, *z* = -2.631, *p* = 0.009). In terms of effect size measures, r-squared values showed that the proportion of variance explained by the covariates ranged between 19-21% for the intercept and slope of language. Additionally, it was near 6% for the intercept and 5% to 9% for the pre and postknot slopes of alpha power, respectively. No other covariate effects were significantly different from zero.

The alpha power to non-social stimuli and language multivariate LGCM showed an adequate fit to the data (χ^2^(26) = 45.317, p = 0.011, CFI = 0.932, RMSEA = 0.070 [0.029-0.106], SRMR = 0.041). The results were largely consistent with those from the main model, with the intercept factors not significantly predicting the cross-domain slope factors (all *ps* > 0.1). Males showed lower initial language scores than females (Estimate = -0.456, *SE* = 0.115, *z* = -3.995, *p* < 0.001), but did not differ in the rate of change in language scores over time. Maternal education was a marginally significant predictor of language ability at baseline (Estimate = 0.135, *SE* = 0.073, *z* = 1.838, *p* = 0.066) and a highly significant predictor of developmental change in language ability (Estimate = 0.323, *SE* = 0.112, *z* = 2.894, *p* = 0.004). Last, the autism likelihood group became a marginally nonsignificant predictor of the slope of language (Estimate = -0.349, *SE* = 0.181, *z* = -1.929, *p* = 0.054). In addition, the autism likelihood group remained a marginally significant predictor of alpha power at the baseline (Estimate = 0.017, *SE* = 0.010, *z* = 1.777, *p* = 0.076). The r-squared values showed that the proportion of variance explained by the covariates was around 19-20% for the intercept and slope of language. Additionally, it was near 4% for the intercept and 3% and 9% for the pre and postknot slopes of alpha power, respectively. No other covariate effects were significantly different from zero.

**Supporting Information 9. EEG Power Differences Between Social and Non-Social Conditions and Links to Language**

We conducted further supplementary analysis to examine whether EEG power differences between social and non-social conditions (social – non-social condition) were related to language development. In the theta power model, due to large differences in the scaling of the EEG and language variables, the condition contrasts were initially rescaled to aid with model interpretation and convergence.

The theta power LGCM fitted the data well (χ^2^(26) = 35.334, p = 0.105, CFI = 0.950, RMSEA = 0.061 [0.000-0.109], SRMR = 0.052). The results did not reveal significant relationships between the intercept factors and the cross-domain slope factors (all ps > 0.2). Males showed lower initial scores on language skills than females (Estimate = -0.455, *SE* = 0.115, *z* = -3.975, *p* < 0.001) but did not show any associations in the rate of change in language scores over time. Further, males showed less steep increases in the postknot slope of theta power than females (Estimate = -0.228, *SE* = 0.107, *z* = -2.133, *p* = 0.033). Maternal education was a highly significant predictor of developmental change in language ability (Estimate = 0.339, *SE* = 0.108, *z* = 3.146, *p* = 0.002). There was a negative covariance between the intercept and postknot slope of theta power ability (Estimate = -0.187, *SE* = 0.073, *z* = -2.549, *p* = 0.011). The r-squared values showed that the proportion of variance explained by the covariates ranged between 20-25% for the intercept and slope of language. Additionally, it was near 4% for the intercept and 2% for the pre and postknot slopes of theta power. No other covariate effects were significantly different from zero.

Initial run of the alpha power model led to convergence and estimation issues. Rescaling the EEG variables and redefining the piecewise slope loadings led to model convergence. The alpha power slope and intercept factors were redefined such that they modelled relative change in standardized alpha power (mean = 0, *SD* =1). The preknot and postknot slope represented change from 10 to 24 months and 24 to 36 months, respectively. The alpha power model fitted the data well (χ^2^(26) = 28.088, p = 0.354, CFI = 1.000, RMSEA = 0.023 [0.000-0.69], SRMR = 0.040). The results showed nonsignificant associations between the intercept factors and the cross-domain slope factors (all ps > 0.6). Consistent with the main model, males showed lower scores on language skills than females at baseline (Estimate = -0.456, *SE* = 0.115, *z* = -3.974, *p* < 0.001), but did not differ in the rate of change in language scores. Maternal education remained a marginally significant predictor of language ability at baseline (Estimate = 0.136, *SE* = 0.074, *z* = 1.846, *p* = 0.065) and a highly significant predictor of developmental change in language ability (Estimate = 0.335, *SE* = 0.108, *z* = 3.100, *p* = 0.002). The autism likelihood group became a marginally nonsignificant predictor of the slope of language, (Estimate = -0.335, *SE* = 0.179, *z* = -1.867, *p* = 0.062). Furthermore, a change in alpha power modulation to social vs. non-social stimuli at 24-36 months negatively covaried with the slope of language (Estimate = -0.196, *SE* = 0.096, *z* = -2.028, *p* = 0.043), meaning that children who showed a stronger neural contrast between social and non-social stimuli at 36 months tended to show less growth in language over time. The r-squared values showed that the proportion of variance explained by the covariates were 20% for the intercept and 19% for the slope of language. Additionally, it was 5% for the intercept, 2% for the preknot and 9% for the postknot slopes of alpha power. No other covariate effects were significantly different from zero.

**Supporting Information 10. Examining Lateralisation Effects**

Considering previously reported language-related lateralisation effects, we conducted supplementary analysis to examine whether the identified relationship between theta power and language was driven by lateralisation effects.

As a first step, we averaged theta power to social and non-social stimuli at each time point for the left and right hemisphere separately. We subsequently calculated a lateralisation index (LI) at each time point, which reflected the relative balance of EEG power between the left and right frontal regions. The LI was computed by taking the difference between left and right frontal theta and alpha power to social and nonsocial stimuli, divided by their sum, resulting in a normalised value ranging from −1 to +1. Positive values indicated greater left-hemisphere activity, while negative values indicated greater right-hemisphere activity. This index allowed us to capture individual differences in the direction and degree of brain asymmetry across development.

Theta power values were rescaled for modelling purposes; the variances of theta power LI were brought into a similar scale range to the variances of language by multiplying theta power by 100. This did not affect the interpretation of the results. The theta power LI and language multivariate LGCM fitted the data well (χ^2^(26) = 35.277, p = 0.106, CFI = 0.942, RMSEA = 0.058 [0.000-0.109], SRMR = 0.061). There was a significant negative covariance between the intercept and preknot slope of the lateralisation index (Estimate = 0.736, *SE* = 3.815, *z* = -2.999, *p* = 0.003), suggesting that infants who were more left-lateralised at baseline showed smaller changes in lateralisation between 10-36 months. Additionally, there was a negative covariance between the preknot and postknot slopes (Estimate = -0.678, *SE* = 1.135, *z* = -2.137, *p* = 0.033), with initial changes in lateralisation slowing across time. Maternal education significantly predicted developmental change in language (Estimate = 0.333, *SE* = 0.109, *z* = 3.030, *p* = 0.002). No significant across-domain relationships were observed between lateralisation and language, suggesting that within this sample, individual differences in brain asymmetry were not predictive of language development trajectories and vice versa.

**Supporting Information 11. Examining EEG power in Relation to Expressive and Receptive Language**

Considering previous evidence linking EEG power and expressive language at earlier time points (Huberty et al., 2023; Levin et al., 2017), a supplementary analysis was undertaken to explore whether such associations were present in the currently study but potentially masked by averaging receptive and expressive language scores. Thus, the analysis was repeated examining receptive and expressive language separately in relation to theta and alpha power.

The theta power and expressive language model fitted the data well (χ^2^(26) = 38.372, p = 0.056, CFI = 0.956, RMSEA = 0.064 [0.000-0.093], SRMR = 0.042). No significant associations were observed between the intercept factors and the cross-domain slope factors (all *ps* > 0.1). The covariance between the intercepts and slopes of theta power and expressive language was nonsignificant (*ps* > 0.1). In addition, the theta power and receptive language model provided an excellent fit to the data (χ^2^(26) = 22.453, p = 0.664, CFI = 1.000, RMSEA = 0.000 [0.000-0.062], SRMR = 0.036). The intercept of theta power was a significant negative predictor of the slope of receptive language (Estimate = -6.070, *SE* = 2.075, *z* = -2.925, *p* = 0.003), indicating that the identified associations between theta power and language in the main model were driven by receptive language. No other cross-domain associations were observed.

The alpha power and expressive language multivariate LGCM demonstrated an adequate fit to the data (χ^2^(26) = 52.373, p = 0.002, CFI = 0.91, RMSEA = 0.081 [0.045-0.116], SRMR = 0.043). The relationship between the intercept factors and the cross-domain slope factors was not significant (all *ps* > 0.1). Similarly, the covariance between the intercepts and slopes of alpha power and expressive language was nonsignificant (*ps* > 0.1). Furthermore, the model examining alpha power in relation to receptive language provided a good fit to the data (χ^2^(26) = 33.163, p = 0.157, CFI = 0.969, RMSEA = 0.053 [0.000-0.097], SRMR = 0.036). The results mirrored those of the alpha power and expressive language model, with negligible associations observed between alpha power and receptive language (all *ps* > 0.1).

**Supporting Information 12. Evaluating Theta Power Links with Non-Verbal MSEL Domains**

To examine whether the theta power and language development association was specific to language or reflected general cognitive development, we tested if similar relationships existed with the fine motor and visual reception domains of the MSEL. Since these variables had not been included in the main model, we conducted LGCMs to evaluate their functional form of growth before running the main analysis.

Attempts to evaluate the functional form of growth for visual reception resulted in model estimation failure. Therefore, this variable was excluded from further analysis. In terms of the fine motor domain, the intercept-only model provided a poor fit to the data. Expanding the model to include a linear slope factor did not substantially improve fit, with most fit indices indicating poor model fit. As residual variances were unequal across time points, we did not impose equality constraints on the residuals. The quadratic growth model also demonstrated poor fit. Additionally, attempts to estimate a piecewise linear growth model resulted in estimation issues, suggesting that this model may not be suitable for the current data. The latent basis model provided an acceptable fit to the data and did not present any estimation issues. Therefore, it was selected as the one that best represented the functional form of growth for fine motor skills (χ^2^(3) = 7.845, *p* = 0.049, CFI = 0.954, RMSEA = 0.084 [0.000-0.191], SRMR = 0.055). **Table S15** provides the results of model fit of the fine motor LGCM.

The theta power and fine motor development model fitted the data well (χ^2^(26) = 29.494, p = 0.289, CFI = 0.995, RMSEA = 0.018 [0.000-0.078], SRMR = 0.042). No significant associations were observed between the intercept of theta power and the fine motor slope factor (Estimate = -4.741, *SE* = 2.532, *z* = -1.872, *p* = 0.061), indicating that the association was more specific to language than general cognitive development in the current sample. No other cross-domain associations were observed.

| **Table S1.**  *Comparison of Trial Counts Across Likelihood and Outcome Groups at Each Age and Condition* | | | | | |
| --- | --- | --- | --- | --- | --- |
| **Age (mo)** | **Condition** | **Group comparison** | ***M* (SD)** | ***t* / *F* (df)** | ***p*-value** |
| 10 | Face | TL vs. EL | TL = 96.22 (42.98) EL = 98.57 (46.25) | -0.3 (137) | 0.768 |
|  |  | TL vs. EL-no-autism vs. EL-autism | TL = 96.22 (42.98) | 0.96 (2) | 0.385 |
|  |  |  | EL-no autism = 101.03 (45.64) |  |  |
|  |  |  | EL-autism = 81.39 (49.11) |  |  |
| 10 | Toy | TL vs. EL | TL = 92.37 (39.24) EL = 87.86 (40.93) | 0.64 (137) | 0.526 |
|  |  | TL vs. EL-no-autism vs. EL-autism | TL = 92.37 (39.24) | 0.47 (2) | 0.624 |
|  |  |  | EL-no autism = 86.66 (41.57) |  |  |
|  |  |  | EL-autism = 96.26 (36.73) |  |  |
| 14 | Face | TL vs. EL | TL = 122.59 (48.1) EL = 121.86 (44.48) | 0.08 (108) | 0.936 |
|  |  | TL vs. EL-no-autism vs. EL-autism | TL = 122.59 (48.1) | 1.03 (2) | 0.359 |
|  |  |  | EL-no autism = 119.09 (45.90) |  |  |
|  |  |  | EL-autism = 143.65 (22.66) |  |  |
| 14 | Toy | TL vs. EL | TL = 110.36 (46.75) EL = 44.02 (5.22) | 0.29 (108) | 0.772 |
|  |  | TL vs. EL-no-autism vs. EL-autism | TL = 110.36 (46.75) | 0.80 (2) | 0.45 |
|  |  |  | EL-no autism = 105.41 (45.21) |  |  |
|  |  |  | EL-autism = 126.20 (29.04) |  |  |
| 24 | Face | TL vs. EL | TL = 130.57 (40.21) EL = 130.34 (40.59) | 0.03 (103) | 0.977 |
|  |  | TL vs. EL-no-autism vs. EL-autism | TL = 130.57 (40.21) | 0.05 (2) | 0.954 |
|  |  |  | EL-no autism = 129.72 (40.65) |  |  |
|  |  |  | EL-autism = 134.18 (42.45) |  |  |
| 24 | Toy | TL vs. EL | TL = 121.68 (41.99) EL = 120.12 (44.59) | 0.18 (103) | 0.859 |
|  |  | TL vs. EL-no-autism vs. EL-autism | TL = 121.68 (41.99) | 0.02 (2) | 0.982 |
|  |  |  | EL-no autism = 120.28 (44.64) |  |  |
|  |  |  | EL-autism = 119.13 (46.98) |  |  |
| 36 | Face | TL vs. EL | TL = 116.70 (43.28) EL = 119.32 (40.70) | -0.32 (105) | 0.753 |
|  |  | TL vs. EL-no-autism vs. EL-autism | TL = 116.70 (43.28) | 1.24 (2) | 0.293 |
|  |  |  | EL-no autism = 116.19 (41.77) |  |  |
|  |  |  | EL-autism = 139.13 (27.07) |  |  |
| 36 | Toy | TL vs EL | TL = 114.64 (39.68) EL = 112.78 (36.56) | 0.25 (105) | 0.805 |
|  |  | TL vs. EL-no-autism vs. EL-autism | TL = 114.64 (39.68) | 0.77 (2) | 0.464 |
|  |  |  | EL-no autism = 110.54 (37.62) |  |  |
|  |  |  | EL-autism = 127.01 (26.33) |  |  |

| **Table S2.** | | |
| --- | --- | --- |
| *Mean receptive, expressive and combined expressive and receptive language raw scores at 10, 14, 24 and 36 months across the whole sample* | | |
| **Variables** | **N** | **Mean (SD)** |
| Receptive language 10 | 152 | 9.34 (2.55) |
| Receptive language 14 | 142 | 11.83 (2.88) |
| Receptive language 24 | 128 | 25.44 (4.70) |
| Receptive language 36 | 90 | 41.18 (8.99) |
| Expressive language 10 | 152 | 8.18 (2.71) |
| Expressive language 14 | 142 | 11.56 (3.32) |
| Expressive language 24 | 129 | 23.46 (5.53) |
| Expressive language 36 | 90 | 38.53 (9.47) |
| Averaged language 10 | 152 | 8.76 (2.19) |
| Averaged language 14 | 142 | 11.69 (2.58) |
| Averaged language 24 | 129 | 24.43 (4.67) |
| Averaged language 36 | 90 | 39.86 (8.63) |
| *Note.* Averaged language: Sum of receptive and expressive language raw scores. | | |

| **Table S3.**  *Results of the Shapiro-Wilk Tests Assessing the Normality of the Distribution of Scores of the Variables Used in the LGCMs* | | |
| --- | --- | --- |
| **Variable** | ***W*** | ***p*** |
| Language 10 | 0.957 | 0.002** |
| Language 14 | 0.967 | 0.012* |
| Language 24 | 0.977 | 0.076 |
| Language 36 | 0.966 | 0.009** |
| Theta power 10 | 0.975 | 0.182 |
| Theta power 14 | 0.992 | 0.928 |
| Theta power 24 | 0.968 | 0.078 |
| Theta power 36 | 0.991 | 0.903 |
| Alpha power 10 | 0.943 | 0.004** |
| Alpha power 14 | 0.972 | 0.132 |
| Alpha power 24 | 0.859 | <0.001*** |
| Alpha power 36 | 0.866 | <0.001*** |
| * p < 0.05. ** p < 0.1. *** p <0.001.  *Abbreviations*: W: Shapiro-Wilk test; p: p-value of the Shapiro-Wilk statistic. | | |

| **Table S4.**  *Order of The Latent Growth Curve Models (LGCMs) Used in the Main Analysis* | | | | |
| --- | --- | --- | --- | --- |
| **Models** | **Variables Modelled** | **Purpose** | **Covariates Included?** | |
| Univariate LGCM - Language | Language | Identify best-fitting growth model | | No |
| Univariate LGCM - Theta | Theta power | Identify best-fitting growth model | | No |
| Univariate LGCM - Alpha | Alpha power | Identify best-fitting growth model | | No |
| Multivariate LGCM - Language & Theta | Language & Theta power | Test intercept/slope associations across domains | | No |
| Multivariate LGCM - Language & Alpha | Language & Alpha power | Test intercept/slope associations across domains | | No |
| Conditional Multivariate LGCM - Theta & Language | Language & Theta power | Test cross-domain associations controlling for sex, maternal education, autism likelihood group | | Yes |
| Conditional Multivariate LGCM -Alpha & Language | Language & Alpha power | Test cross-domain associations controlling for sex, maternal education, autism likelihood group | | Yes |

| **Table S5.**  *Latent Growth Curve Modelling of Language: Model Fit Results Testing the Functional Forms of Growth of the Language Model* | | | | | | | |  |
| --- | --- | --- | --- | --- | --- | --- | --- | --- |
|  | **Parameters** | **Log-likelihood** | **AIC** | **BIC** | **χ^2^ value (df)** | **CFI** | **RMSEA [90%]** | **SRMR** |
| Intercept-only model | 6 | -2136.556 | 4285.111 | 4303.524 | 351.270 (8), *p* < 0.001 | 0.000 | 0.626 [0.575-0.678] | 0.905 |
| Linear growth model^a^ | 9 | -2002.360 | 4020.721 | 4045.272 | 135.272 (6), p < 0.001 | 0.207 | 0.388 [0.332-0.446] | 0.249 |
| Quadratic growth model | 13 | -1995.299 | 4016.599 | 4056.494 | 89.706 (1), p < 0.001 | 0.276 | 0.907 [0.768-1.055] | 0.271 |
| Piecewise linear growth model | 13 | -1963.582 | 3953.163 | 3993.059 | 26.681 (1), p <0.001 | 0.842 | 0.402 [0.306-0.507] | 0.126 |
| Latent basis growth model | 11 | -1946.092 | 3914.184 | 3947.942 | 12.138 (3), p = 0.007 | 0.942 | 0.138 [0.066-0.242] | 0.060 |
| ^a^Initial run of the model resulted in negative variance estimate for the 'language’ observed variable at 36 months. The variance was manually fixed to 0. | | | | | | | | |
| *Abbreviations*. AIC: Akaike’s Information Criterion; BIC: Bayesian Information Criterion: χ^2^: chi-square value; CFI: comparative fit index; RMSEA: root mean square error of approximation; SRMR: standardized root mean square residual. | | | | | | | | |

| **Table S6.**  *Parameter Estimates of the Univariate Language Latent Basis Model* | | | | | |
| --- | --- | --- | --- | --- | --- |
| ***Latent variables*** | **Estimate** | ***SE*** | ***z*** | ***p*** | **Std.all** |
| Int. Lang 10 | 1 | - | - | - | 0.631 |
| Int. Lang 14 | 1 | - | - | - | 0.753 |
| Int. Lang 24 | 1 | - | - | - | 0.515 |
| Int. Lang 36 | 1 | - | - | - | 0.525 |
| Slp. Lang 10 | 0 | - | - | - | 0.000 |
| Slp. Lang 14 | -0.139 | 0.046 | -2.996 | 0.003 | -0.132 |
| Slp. Lang 24 | 0.835 | 0.046 | 18.001 | <0.001 | 0.544 |
| Slp. Lang 36 | 1 | - | - | - | 0.665 |
| ***Mean*** | **Estimate** | ***SE*** | ***z*** | ***p*** | **Std.all** |
| Intercept | 36.64 | 0.722 | 50.755 | <0.001 | 6.145 |
| Slope | 17.669 | 1.019 | 17.348 | <0.001 | 2.342 |
| ***Variance*** | **Estimate** | ***SE*** | ***z*** | ***p*** | **Std.all** |
| Lang 10 | 53.818 | 7.916 | 6.799 | <0.001 | 0.602 |
| Lang 14 | 27.184 | 6.832 | 3.979 | <0.001 | 0.433 |
| Lang 24 | 52.386 | 10.415 | 5.030 | <0.001 | 0.391 |
| Lang 36 | 28.501 | 10.955 | 2.602 | 0.009 | 0.221 |
| Intercept | 35.547 | 6.916 | 5.140 | <0.001 | 1 |
| Slope | 56.921 | 12.889 | 4.416 | <0.001 | 1 |
|  | **Estimate** | ***SE*** | ***z*** | ***p*** | **Std.all** |
| *Factor covariance* | 3.882 | 7.571 | 0.513 | 0.608 | 0.086 |
| *Abbreviations.* SE: Standard error; Lang: Language; Int. Lang: Intercept of language; Slp. Lang: Slope of language. | | | | | |

| **Table S7.**  *Latent Growth Curve Modelling of Theta Power: Model Fit Results Testing the Functional Forms of Growth of the Theta Power Model* | | | | | | | |  |
| --- | --- | --- | --- | --- | --- | --- | --- | --- |
|  | **Parameters** | **Log-likelihood** | **AIC** | **BIC** | **χ^2^ value (df)** | **CFI** | **RMSEA [90%]** | **SRMR** |
| Intercept-only model | 6 | 594.449 | -1176.898 | -1158.637 | 196.128 (8), p < 0.001 | 0.000 | 0.463 [0.405-0.524] | 0.391 |
| Linear growth modela | 9 | 684.335 | -1350.670 | -1323.279 | 21.324 (5), p = 0.001 | 0.891 | 0.172 [0.099-0.254] | 0.077 |
| Quadratic growth model | 13 | 694.357 | -1362.713 | -1323.149 | 0.441 (1), p = 0.507 | 1 | 0.000 [0.000-0.226] | 0.011 |
| Piecewise linear growth modelb | 13 | 693.350 | -1366.700 | -1336.265 | 5.881 (4), p = 0.208 | 0.994 | 0.045 [0.000-0.120] | 0.022 |
| Latent basis growth model | 11 | 694.448 | -1366.897 | -1333.419 | 0.295 (3), p = 0.961 | 1 | 0.000 [0.000-0.000] | 0.012 |
| *Abbreviations*. AIC: Akaike’s Information Criterion; BIC: Bayesian Information Criterion: χ^2^: chi-square value; CFI: comparative fit index; RMSEA: root mean square error of approximation; SRMR: standardized root mean square residual. | | | | | | | | |

| **Table S8.**  *Parameter Estimates of the Univariate Theta Power Piecewise Linear Model* | | | | | |
| --- | --- | --- | --- | --- | --- |
| ***Mean*** | **Estimate** | ***SE*** | ***z*** | ***p*** | **Std.all** |
| Intercept | 0.719 | 0.005 | 136.19 | <0.001 | 14.047 |
| Preknot slope | -0.035 | 0.005 | -6.500 | <0.001 | -1.261 |
| Postknot slope | -0.011 | 0.001 | -9.046 | <0.001 | -1.452 |
| ***Variance*** | **Estimate** | ***SE*** | ***z*** | ***p*** | **Std.all** |
| Theta 10 | 0.001 | 0.000 | 4.513 | <0.001 | 0.336 |
| Theta 14 | 0.001 | 0.001 | 2.300 | 0.021 | 0.319 |
| Theta 24 | 0.001 | 0.000 | 4.153 | <0.001 | 0.367 |
| Theta 36 | 0.001 | 0.000 | 2.743 | 0.006 | 0.343 |
| Intercept | 0.003 | 0.001 | 4.862 | <0.001 | 1 |
| Preknot slope | 0.001 | 0.001 | 0.815 | 0.415 | 1 |
| Postknot slope | 0.000 | 0.000 | 1.497 | 0.134 | 1 |
| ***Factor covariance*** | **Estimate** | ***SE*** | ***z*** | ***p*** | **Std.all** |
| Int. Theta & preknot slope | 0.000 | 0.000 | -0.626 | 0.531 | -0.198 |
| Int. Theta & postknot slope | 0.000 | 0.000 | -1.608 | 0.108 | -0.351 |
| Preknot & postknot slopes | 0.000 | 0.000 | -0.285 | 0.776 | -0.191 |
| *Abbreviations.* SE: Standard error; Int. Theta: Intercept of theta power. | | | | | |

| **Table S9.**  *Latent Growth Curve Modelling of Alpha Power: Model Fit Results Testing the Functional Forms of Growth of the Alpha Power Model* | | | | | | | |  |
| --- | --- | --- | --- | --- | --- | --- | --- | --- |
|  | **Parameters** | **Log-likelihood** | **AIC** | **BIC** | **χ^2^ value (df)** | **CFI** | **RMSEA [90%]** | **SRMR** |
| Intercept-only model | 6 | 752.295 | -1492.591 | -1474.330 | 56.403 (8), p <0.001 | 0.587 | 0.269 [0.202-0.340] | 0.243 |
| Linear growth model^a^ | 9 | 771.430 | -1524.861 | -1497.470 | 26.443 (5), p <0.001 | 0.809 | 0.231 [0.151-0.321] | 0.109 |
| Quadratic growth model | 13 | 784.274 | -1542.548 | -1502.984 | 1.490 (1), p = 0.222 | 0.963 | 0.228 [0.082-0.417] | 0.046 |
| Piecewise linear growth model^b^ | 13 | 783.224 | -1546.448 | -1516.014 | 20.584 (4), p <0.001 | 0.942 | 0.142 [0.082-0.209] | 0.041 |
| Latent basis growth model^c^ | 10 | 785.834 | -1551.668 | -1521.233 | 2.302 (4), p = 0.680 | 1 | 0.000 [0.000-0.155] | 0.037 |
| ^a^The model was also run constraining the residuals to equality which resulted in poor model fit (χ2(8) = 63.826, p <0.001, CFI = 0.594, RMSEA = 0.266, SRMR = 0.138). | | | | | | | | |
| ^b^Piecewise linear growth model with homoschedastic errors. | | | |  |  |  |  |  |
| ^c^Initial run of the model resulted in negative variance estimate for the alpha power observed variable at 10 months. The variance was manually fixed to 0. | | | | | | | | |
| *Abbreviations*. AIC: Akaike’s Information Criterion; BIC: Bayesian Information Criterion: χ^2^: chi-square value; CFI: comparative fit index; RMSEA: root mean square error of approximation; SRMR: standardized root mean square residual. | | | | | | | | |

| **Table S10.**  *Parameter Estimates of the Univariate Alpha Power Piecewise Linear Model* | | | | | |
| --- | --- | --- | --- | --- | --- |
| ***Mean*** | **Estimate** | ***SE*** | ***z*** | ***p*** | **Std.all** |
| Intercept | 0.157 | 0.004 | 35.932 | <0.001 | 3.447 |
| Preknot slope | 0.027 | 0.005 | 5.259 | <0.001 | 0.612 |
| Postknot slope | 0.001 | 0.001 | 1.047 | 0.295 | 0.126 |
| ***Variance*** | **Estimate** | ***SE*** | ***z*** | ***p*** | **Std.all** |
| Alpha 10 | 0.001 | 0.000 | 2.465 | 0.014 | 0.215 |
| Alpha 14 | 0.001 | 0.000 | 1.355 | 0.175 | 0.189 |
| Alpha 24 | 0.001 | 0.000 | 3.508 | <0.001 | 0.258 |
| Alpha 36 | 0.001 | 0.000 | 1.281 | 0.200 | 0.222 |
| Intercept | 0.002 | 0.000 | 4.759 | <0.001 | 1 |
| Preknot slope | 0.002 | 0.001 | 2.283 | 0.022 | 1 |
| Postknot slope | 0.000 | 0.000 | 2.57 | 0.010 | 1 |
| ***Factor covariance*** | **Estimate** | ***SE*** | ***z*** | ***p*** | **Std.all** |
| Int. Alpha & preknot slope | -0.001 | 0.001 | -1.591 | 0.112 | -0.400 |
| Int. Alpha & postknot slope | -0.001 | 0.000 | -1.617 | 0.106 | -0.311 |
| Preknot & postknot slopes | 0.000 | 0.000 | -1.049 | 0.294 | -0.316 |
| *Abbreviations.* SE: Standard error; Int. Alpha: Intercept of alpha power. | | | | | |

| **Table S11.**  *Parameter Estimates of the Theta Power and Language Multivariate Latent Growth Curve Model* | | | | | | |
| --- | --- | --- | --- | --- | --- | --- |
| **Regressions** | **Predictors** | **Estimate** | ***SE*** | ***z*** | ***p*** | **Std.all** |
| Slope lang | Int. Theta | -3.961 | 2.177 | -1.819 | 0.069 | -0.270 |
| Preknot theta | Int. Lang | -0.007 | 0.012 | -0.528 | 0.598 | -0.137 |
| Postknot theta | Int. Lang | 0.002 | 0.002 | 0.794 | 0.427 | 0.148 |
| **Covariances** | **Covariates** | **Estimate** | ***SE*** | ***z*** | ***p*** | **Std.all** |
| Int. Lang | Int. Theta | -0.001 | 0.004 | -0.318 | 0.750 | -0.038 |
| Int. Lang | Slope lang | 0.039 | 0.074 | 0.530 | 0.596 | 0.091 |
| Slope lang | Preknot theta | 0.009 | 0.006 | 1.565 | 0.118 | 0.441 |
| Slope lang | Postknot theta | -0.001 | 0.001 | -1.128 | 0.259 | -0.260 |
| Int. Theta | Preknot theta | -0.000 | 0.000 | -0.608 | 0.543 | -0.196 |
| Int. Theta | Postknot theta | -0.000 | 0.000 | -1.595 | 0.111 | -0.355 |
| Preknot theta | Postknot theta | -0.000 | 0.000 | -0.249 | 0.804 | -0.168 |
| *Abbreviations*. SE: Standard error; Lang: Language; Int. Lang: Intercept of language; Int. Theta: Intercept of theta power. | | | | | | |

| **Table S12.**  *Parameter Estimates of the Alpha Power and Language Multivariate Latent Growth Curve Model* | | | | | | |
| --- | --- | --- | --- | --- | --- | --- |
| **Regressions** | **Predictors** | **Estimate** | ***SE*** | ***z*** | ***p*** | **Std.all** |
| Slope lang | Int. Alpha | 1.512 | 2.304 | 0.656 | 0.512 | 0.092 |
| Preknot alpha | Int. Lang | -0.001 | 0.011 | -0.110 | 0.912 | -0.016 |
| Postknot alpha | Int. Lang | -0.002 | 0.002 | -0.802 | 0.423 | -0.107 |
| **Covariances** | **Covariates** | **Estimate** | ***SE*** | ***z*** | ***p*** | **Std.all** |
| Int. Lang | Int. Alpha | 0.003 | 0.003 | 1.015 | 0.310 | 0.11 |
| Int. Lang | Slope lang | 0.036 | 0.075 | 0.477 | 0.634 | 0.080 |
| Slope lang | Preknot alpha | -0.007 | 0.005 | -1.342 | 0.180 | -0.215 |
| Slope lang | Postknot alpha | 0.001 | 0.001 | 0.420 | 0.675 | 0.074 |
| Int. Alpha | Preknot alpha | -0.001 | 0.001 | -1.557 | 0.119 | -0.395 |
| Int. Alpha | Postknot alpha | -0.000 | 0 | -1.564 | 0.118 | -0.296 |
| Preknot alpha | Postknot alpha | -0.000 | 0 | -1.110 | 0.267 | -0.332 |
| *Abbreviations*. SE: Standard error; Lang: Language; Int. Lang: Intercept of language; Int. Alpha: Intercept of alpha power. | | | | | | |

| **Table S13.**  *Parameter Estimates of the Theta Power and Language Multivariate Latent Growth Curve Model controlling for Autism Llikelihood Group, Biological Sex and Maternal Education* | | | | | | |
| --- | --- | --- | --- | --- | --- | --- |
| **Regressions** | **Predictors** | **Estimate** | ***SE*** | ***z*** | ***p*** | **Std.all** |
| Slope lang | Int. Theta | -4.435 | 2.121 | -2.091 | 0.037 | -0.304 |
| Preknot theta | Int. Lang | -0.018 | 0.016 | -1.109 | 0.267 | -0.372 |
| Postknot theta | Int. Lang | 0.004 | 0.003 | 1.384 | 0.166 | 0.334 |
| Int. Lang | Group | -0.156 | 0.121 | -1.295 | 0.195 | -0.128 |
| Int. Lang | Sex | -0.455 | 0.115 | -3.953 | <0.001 | -0.381 |
| Int. Lang | Maternal education | 0.133 | 0.077 | 1.733 | 0.083 | 0.174 |
| Slope lang | Group | -0.408 | 0.168 | -2.422 | 0.015 | -0.267 |
| Slope lang | Sex | -0.037 | 0.163 | -0.227 | 0.820 | -0.025 |
| Slope lang | Maternal education | 0.300 | 0.106 | 2.819 | 0.005 | 0.314 |
| Int. Theta | Group | -0.015 | 0.011 | -1.342 | 0.180 | -0.147 |
| Int. Theta | Sex | 0.002 | 0.011 | 0.176 | 0.860 | 0.018 |
| Int. Theta | Maternal education | -0.005 | 0.007 | -0.772 | 0.440 | -0.084 |
| Preknot theta | Group | -0.006 | 0.014 | -0.410 | 0.682 | -0.098 |
| Preknot theta | Sex | -0.012 | 0.013 | -0.911 | 0.362 | -0.218 |
| Preknot theta | Maternal education | 0.013 | 0.008 | 1.58 | 0.114 | 0.363 |
| Postknot theta | Group | 0.004 | 0.003 | 1.270 | 0.204 | 0.246 |
| Postknot theta | Sex | 0.004 | 0.003 | 1.427 | 0.153 | 0.282 |
| Postknot theta | Maternal education | -0.000 | 0.002 | -0.158 | 0.875 | -0.034 |
| **Covariances** | **Covariates** | **Estimate** | ***SE*** | ***z*** | ***p*** | **Std.all** |
| Int. Lang | Int. Theta | -0.000 | 0.004 | -0.054 | 0.957 | -0.007 |
| Int. Lang | Slope lang | -0.005 | 0.072 | -0.073 | 0.942 | -0.016 |
| Slope lang | Preknot theta | 0.006 | 0.005 | 1.274 | 0.203 | 0.396 |
| Slope lang | Postknot theta | -0.001 | 0.001 | -1.076 | 0.282 | -0.283 |
| Int. Theta | Preknot theta | -0.000 | 0.000 | -0.680 | 0.497 | -0.241 |
| Int. Theta | Postknot theta | -0.000 | 0.000 | -1.319 | 0.187 | -0.355 |
| Preknot theta | Postknot theta | 0.000 | 0.000 | 0.198 | 0.843 | 0.114 |
| *Abbreviations*. SE: Standard error; Lang: Language; Int. Lang: Intercept of language; Int. Theta: Intercept of theta power. | | | | | | |

| **Table S14.**  *Parameter Estimates of the Alpha Power and Language Multivariate Latent Growth Curve Model controlling for Autism Llikelihood Group, Biological Sex and Maternal Education* | | | | | | |
| --- | --- | --- | --- | --- | --- | --- |
| **Regressions** | **Predictors** | **Estimate** | ***SE*** | ***z*** | ***p*** | **Std.all** |
| Slope lang | Int. Alpha | 1.818 | 2.217 | 0.820 | 0.412 | 0.112 |
| Preknot alpha | Int. Lang | 0.004 | 0.014 | 0.269 | 0.788 | 0.049 |
| Postknot alpha | Int. Lang | -0.004 | 0.003 | -1.317 | 0.188 | -0.226 |
| Int. Lang | Group | -0.153 | 0.121 | -1.262 | 0.207 | -0.125 |
| Int. Lang | Sex | -0.456 | 0.115 | -3.968 | <0.001 | -0.383 |
| Int. Lang | Maternal education | 0.133 | 0.077 | 1.736 | 0.083 | 0.174 |
| Slope lang | Group | -0.381 | 0.172 | -2.212 | 0.027 | -0.250 |
| Slope lang | Sex | -0.032 | 0.164 | -0.198 | 0.843 | -0.022 |
| Slope lang | Maternal education | 0.310 | 0.107 | 2.910 | 0.004 | 0.325 |
| Int. Alpha | Group | 0.017 | 0.009 | 1.781 | 0.075 | 0.177 |
| Int. Alpha | Sex | -0.003 | 0.009 | -0.320 | 0.749 | -0.030 |
| Int. Alpha | Maternal education | 0.006 | 0.006 | 1.076 | 0.282 | 0.103 |
| Preknot alpha | Group | -0.003 | 0.012 | -0.247 | 0.805 | -0.031 |
| Preknot alpha | Sex | 0.005 | 0.013 | 0.387 | 0.698 | 0.054 |
| Preknot alpha | Maternal education | -0.010 | 0.008 | -1.249 | 0.212 | -0.169 |
| Postknot alpha | Group | -0.004 | 0.003 | -1.523 | 0.128 | -0.198 |
| Postknot alpha | Sex | -0.003 | 0.003 | -1.253 | 0.210 | -0.177 |
| Postknot alpha | Maternal education | 0.000 | 0.002 | 0.174 | 0.862 | 0.026 |
| **Covariances** | **Covariates** | **Estimate** | ***SE*** | ***z*** | ***p*** | **Std.all** |
| Int. Lang | Int. Alpha | 0.003 | 0.003 | 0.987 | 0.324 | 0.117 |
| Int. Lang | Slope lang | -0.014 | 0.072 | -0.203 | 0.839 | -0.042 |
| Slope lang | Preknot alpha | -0.005 | 0.004 | -1.219 | 0.223 | -0.183 |
| Slope lang | Postknot alpha | 0.000 | 0.001 | 0.048 | 0.962 | 0.008 |
| Int. Alpha | Preknot alpha | -0.001 | 0.001 | -1.585 | 0.113 | -0.401 |
| Int. Alpha | Postknot alpha | -0.000 | 0.000 | -1.456 | 0.145 | -0.264 |
| Preknot alpha | Postknot alpha | -0.000 | 0.000 | -0.992 | 0.321 | -0.310 |
| *Abbreviations*. SE: Standard error; Lang: Language; Int. Lang: Intercept of language; Int. Alpha: Intercept of alpha power. | | | | | | |

| **Table S15.**  *Latent Growth Curve Modelling of Fine Motor Scores: Model Fit Results Testing the Functional Forms of Growth of the Fine Motor Model* | | | | | | | |  |
| --- | --- | --- | --- | --- | --- | --- | --- | --- |
|  | **Parameters** | **Log-likelihood** | **AIC** | **BIC** | **χ^2^ value (df)** | **CFI** | **RMSEA [90%]** | **SRMR** |
| Intercept-only model | 6 | -2148.957 | 4309.915 | 4328.328 | 32.835 (8), *p* < 0.001 | 0.556 | 0.159 [0.106-0.217] | 0.121 |
| Linear growth model | 9 | -2141.801 | 4301.601 | 4329.221 | 19.050 (5), p = 0.002 | 0.779 | 0.142 [0.074-0.217] | 0.088 |
| Quadratic growth model | 13 | -2136.245 | 4298.491 | 4338.386 | 9.000 (1), p = 0.003 | 0.895 | 0.219 [0.095-0.376] | 0.052 |
| Piecewise linear growth model^a^ | 13 | -2134.436 | 4294.871 | 4334.767 | 1.808 (1), p = 0.179 | 0.985 | 0.071[0.000-0.185] | 0.043 |
| Latent basis growth model | 11 | -2135.419 | 4292.837 | 4326.595 | 7.845(3), p = 0.049 | 0.954 | 0.084[0.000-0.191] | 0.055 |
| ^a^ The Piecewise linear growth model provided a good fit to the data but was not selected due to estimation issues (unable to compute robust CFI and RMSEA values and negative latent variable variances). | | | | | | | | |
| *Abbreviations*. AIC: Akaike’s Information Criterion; BIC: Bayesian Information Criterion: χ^2^: chi-square value; CFI: comparative fit index; RMSEA: root mean square error of approximation; SRMR: standardized root mean square residual. | | | | | | | | |

| **** |
| --- |
| **Figure S1.** *Average Trajectories of Language, Theta Power and Alpha Power by Autism Likelihood Group (TL = Typical Likelihood; EL = Elevated Likelihood).* The values in the x-axis represent the age in months. |

| 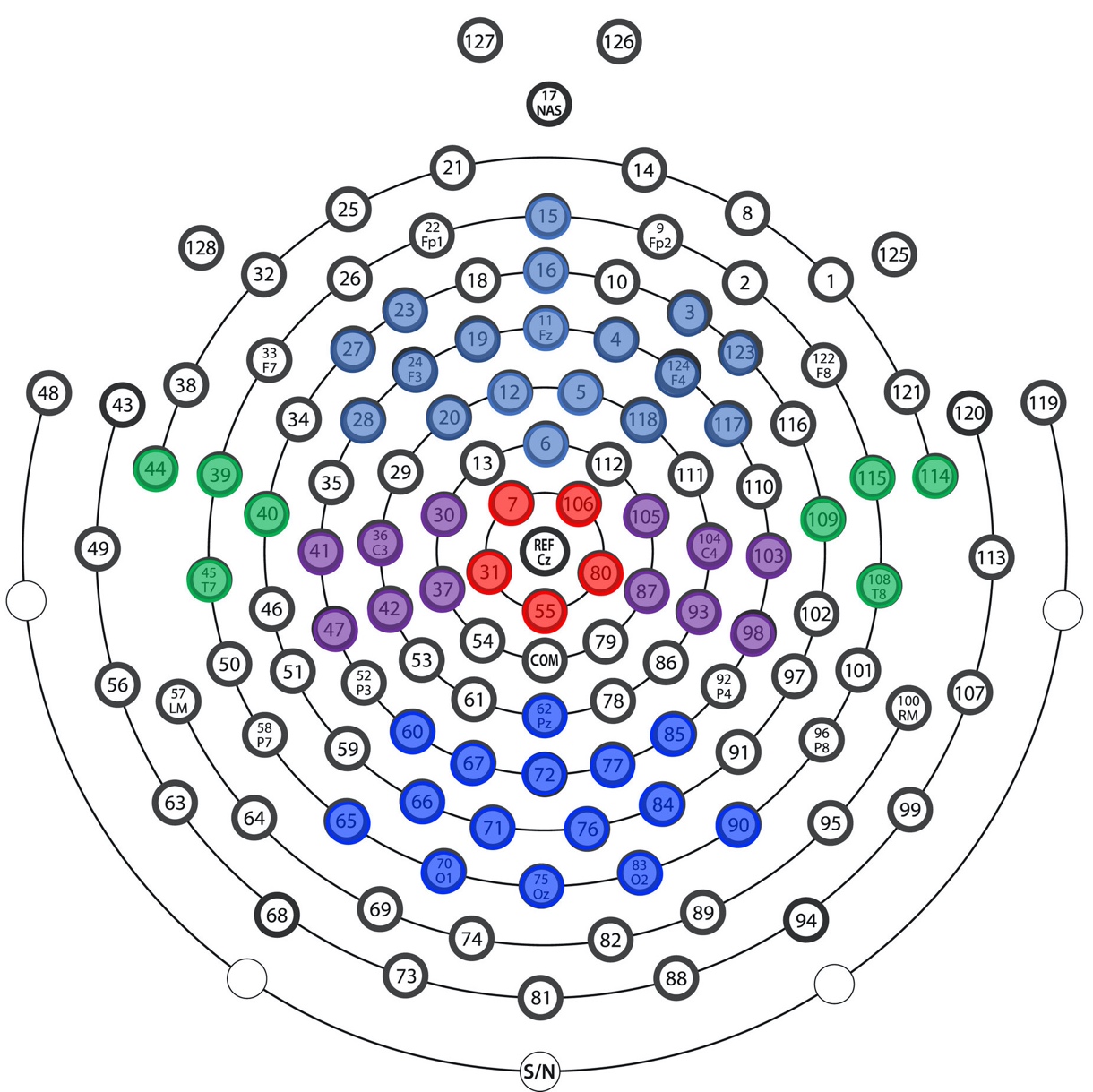 |
| --- |
| **Figure S2.** *Figure Depicting the Groups of Electrodes Used in the Analyses.* Of the electrodes shown, the electrode clusters over the frontal region (pale blue) were used in the analyses. Adapted from Begum-Ali et al. (2022). |

|    |
| --- |
| **Figure S3.** *Individual and averaged power spectra for frontal channels by condition across 10, 14, 24 and 36 months.* Power spectra at 10, 14, 24 and 36 months by individual participant, shown for frontal channels for the social (Face) and non-social (Toy) conditions. The thick black line represents the averaged power spectra; the x-axis represents frequency in Hz; the y-axis indicates relative spectral power values. In all conditions and regions, the alpha and theta peaks are clearly visible within the 3-6 and 6–9 Hz range. |

| A      B |
| --- |
| **Figure S4.** *Histograms Depicting the Frequency Distribution of the Language (S4A), Theta Power and Alpha Power Variables (S4B) at 10, 14, 24 and 36 Months.* |

| **Figure S5**  *Predicted Trajectories of Language Across 10, 14, 24 and 36 months.* |
| --- |
| **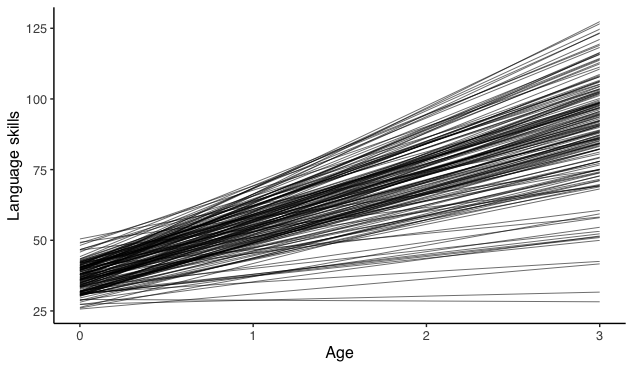** |
| *Note.* The 0, 1, 2 and 3 values of the x-axis represent ages 10, 14, 24 and 36 months. |

| **Figure S6**  *Predicted Trajectories of Theta Power Across 10, 14, 24 and 36 months.* |
| --- |
| **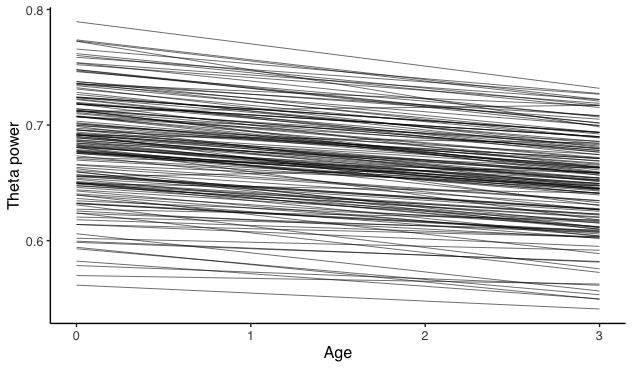** |
| *Note.* The 0, 1, 2 and 3 values of the x-axis represent ages 10, 14, 24 and 36 months. |

| **Figure S7**  *Predicted Trajectories of Alpha Power Across 10, 14, 24 and 36 months.* |
| --- |
| **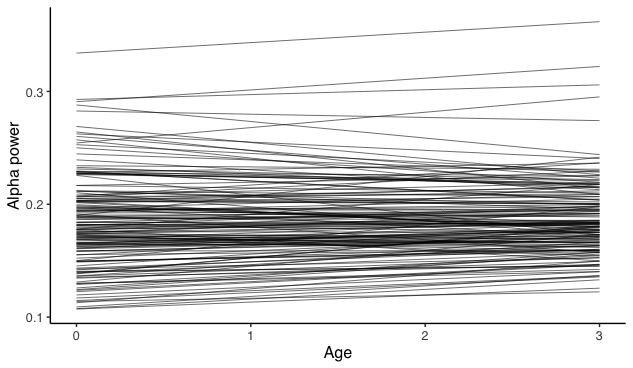** |
| *Note.* The 0, 1, 2 and 3 values of the x-axis represent ages 10, 14, 24 and 36 months. |

**References**

Begum-Ali, J., Goodwin, A., Mason, L., Pasco, G., Charman, T., Johnson, M. H., Jones, E. J. H., & Team, the S. (2022). Altered theta–beta ratio in infancy associates with family history of ADHD and later ADHD-relevant temperamental traits. *Journal of Child Psychology and Psychiatry*, *63*(9), 1057–1067. https://doi.org/10.1111/jcpp.13563

Bentler, P. M., & Bonett, D. G. (1980). Significance tests and goodness of fit in the analysis of covariance structures. *Psychological Bulletin*, *88*(3), 588–606. https://doi.org/10.1037/0033-2909.88.3.588

Burnham, K. P., & Anderson, D. R. (2004). Multimodel Inference: Understanding AIC and BIC in Model Selection. *Sociological Methods & Research*, *33*(2), 261–304. https://doi.org/10.1177/0049124104268644

Hu, L., & Bentler, P. M. (1999). Cutoff criteria for fit indexes in covariance structure analysis: Conventional criteria versus new alternatives. *Structural Equation Modeling: A Multidisciplinary Journal*, *6*(1), 1–55. https://doi.org/10.1080/10705519909540118

Huberty, S., O’Reilly, C., Carter Leno, V., Steiman, M., Webb, S., Elsabbagh, M., & Team, T. B. (2023). Neural mechanisms of language development in infancy. *Infancy*, *28*(4), 754–770. https://doi.org/10.1111/infa.12540

Jones, E. J. H., Mason, L., Begum Ali, J., van den Boomen, C., Braukmann, R., Cauvet, E., Demurie, E., Hessels, R. S., Ward, E. K., Hunnius, S., Bolte, S., Tomalski, P., Kemner, C., Warreyn, P., Roeyers, H., Buitelaar, J., Falck-Ytter, T., Charman, T., & Johnson, M. H. (2019a). Eurosibs: Towards robust measurement of infant neurocognitive predictors of autism across Europe. *Infant Behavior and Development*, *57*, 101316. https://doi.org/10.1016/j.infbeh.2019.03.007

Jones, E. J. H., Mason, L., Begum Ali, J., van den Boomen, C., Braukmann, R., Cauvet, E., Demurie, E., Hessels, R. S., Ward, E. K., Hunnius, S., Bolte, S., Tomalski, P., Kemner, C., Warreyn, P., Roeyers, H., Buitelaar, J., Falck-Ytter, T., Charman, T., & Johnson, M. H. (2019b). Eurosibs: Towards robust measurement of infant neurocognitive predictors of autism across Europe. *Infant Behavior and Development*, *57*, 101316. https://doi.org/10.1016/j.infbeh.2019.03.007

Kenny, D. A., Kaniskan, B., & McCoach, D. B. (2015). The Performance of RMSEA in Models With Small Degrees of Freedom. *Sociological Methods & Research*, *44*(3), 486–507. https://doi.org/10.1177/0049124114543236

Levin, A. R., Varcin, K. J., O’Leary, H. M., Tager-Flusberg, H., & Nelson, C. A. (2017). EEG power at 3 months in infants at high familial risk for autism. *Journal of Neurodevelopmental Disorders*, *9*(1), 34. https://doi.org/10.1186/s11689-017-9214-9

Marsh, H. W., Hau, K.-T., & Wen, Z. (2004). In Search of Golden Rules: Comment on Hypothesis-Testing Approaches to Setting Cutoff Values for Fit Indexes and Dangers in Overgeneralizing Hu and Bentler’s (1999) Findings. *Structural Equation Modeling: A Multidisciplinary Journal*, *11*(3), 320–341. https://doi.org/10.1207/s15328007sem1103_2

McIntosh, C. N. (2007). Rethinking fit assessment in structural equation modelling: A commentary and elaboration on Barrett (2007). *Personality and Individual Differences*, *42*(5), 859–867. https://doi.org/10.1016/j.paid.2006.09.020

Mullen, E. M. (1995). *Mullen scales of early learning*. AGS Circle Pines, MN.

Neath, A. A., & Cavanaugh, J. E. (2012). The Bayesian information criterion: Background, derivation, and applications. *WIREs Computational Statistics*, *4*(2), 199–203. https://doi.org/10.1002/wics.199

Schermelleh-Engel, K., Moosbrugger, H., & Müller, H. (2003). Evaluating the Fit of Structural Equation Models: Tests of Significance and Descriptive Goodness-of-Fit Measures. *Methods of Psychological Research*, *8*, 23–74.

Steiger, J. H. (2007). Understanding the limitations of global fit assessment in structural equation modeling. *Personality and Individual Differences*, *42*(5), 893–898. https://doi.org/10.1016/j.paid.2006.09.017
